# Associations of polymetabolic risk of high maternal pre-pregnancy body mass index with pregnancy complications, birth outcomes, and early childhood neurodevelopment: findings from two pregnancy cohorts

**DOI:** 10.1186/s12884-024-06274-9

**Published:** 2024-01-24

**Authors:** Polina Girchenko, Marius Lahti-Pulkkinen, Esa Hämäläinen, Hannele Laivuori, Pia M. Villa, Eero Kajantie, Katri Räikkönen

**Affiliations:** 1 Department of Psychology and Logopedics, Faculty of Medicine, University of Helsinki, (Haartmaninkatu 3), P.O BOX 21, 00014 Helsinki, Finland; 2 National Institute for Health and Welfare, Helsinki, Finland; 3 Queen’s Medical Research Institute, University of Edinburgh, Edinburgh, UK; 4 Department of Clinical Chemistry, University of Eastern Finland, Kuopio, Finland; 5 Department of Obstetrics and Gynecology, Tampere University Hospital, Tampere, Finland; 6 Center for Child, Adolescent and Maternal Health Research, Faculty of Medicine and Health Technology, Tampere University, Tampere, Finland; 7 Medical and Clinical Genetics, University of Helsinki and Helsinki University Hospital, Helsinki, Finland; 8 Institute for Molecular Medicine Finland, Helsinki Institute of Life Science, University of Helsinki, Helsinki, Finland; 9 Obstetrics and Gynaecology, Helsinki University Hospital and University of Helsinki, Helsinki, Finland; 10 Finnish Institute for Health and Welfare, Public Health Unit, Helsinki, Finland; 11 Clinical Medicine Research Unit, MRC Oulu, University of Oulu and Oulu University Hospital, Oulu, Finland; 12 Department of Clinical and Molecular Medicine, Norwegian University of Science and Technology, Trondheim, Norway

**Keywords:** Pre-pregnancy BMI, BMI-defined metabolome, High BMI-related polymetabolic risk score, Pregnancy complications, Birth outcomes, Childhood neurodevelopment

## Abstract

**Background:**

A substantial proportion of maternal pregnancy complications, adverse birth outcomes and neurodevelopmental delay in children may be attributable to high maternal pre-pregnancy Body Mass Index (BMI). However, BMI alone is insufficient for the identification of all at-risk mothers and children as many women with non-obesity(< 30 kg/m^2^) or normal weight(18.5–24.99 kg/m^2^) and their children may suffer from adversities. Evidence suggests that BMI-related metabolic changes during pregnancy may predict adverse mother–child outcomes better than maternal anthropometric BMI.

**Methods:**

In a cohort of 425 mother–child dyads, we identified maternal BMI-defined metabolome based on associations of 95 metabolic measures measured three times during pregnancy with maternal pre-pregnancy BMI. We then examined whether maternal BMI-defined metabolome performed better than anthropometric BMI in predicting gestational diabetes, hypertensive disorders, gestational weight gain (GWG), Caesarian section delivery, child gestational age and weight at birth, preterm birth, admission to neonatal intensive care unit (NICU), and childhood neurodevelopment. Based on metabolic measures with the highest contributions to BMI-defined metabolome, including inflammatory and glycolysis-related measures, fatty acids, fluid balance, ketone bodies, lipids and amino acids, we created a set of maternal high BMI-related polymetabolic risk scores (PMRSs), and in an independent replication cohort of 489 mother–child dyads tested their performance in predicting the same set of mother–child outcomes in comparison to anthropometric BMI.

**Results:**

BMI-defined metabolome predicted all of the studied mother–child outcomes and improved their prediction over anthropometric BMI, except for gestational hypertension and GWG. BMI-related PMRSs predicted gestational diabetes, preeclampsia, Caesarian section delivery, admission to NICU, lower gestational age at birth, lower cognitive development score of the child, and improved their prediction over anthropometric BMI. BMI-related PMRSs predicted gestational diabetes, preeclampsia, Caesarean section delivery, NICU admission and child’s lower gestational age at birth even at the levels of maternal non-obesity and normal weight.

**Conclusions:**

Maternal BMI-defined metabolome improves the prediction of pregnancy complications, birth outcomes, and neurodevelopment in children over anthropometric BMI. The novel, BMI-related PMRSs generated based on the BMI-defined metabolome have the potential to become biomarkers identifying at-risk mothers and their children for timely targeted interventions even at the level of maternal non-obesity and normal weight.

**Supplementary Information:**

The online version contains supplementary material available at 10.1186/s12884-024-06274-9.

## Background

Maternal pre-pregnancy obesity (Body Mass Index [BMI] ≥ 30 kg/m^2^) has become a major challenge of obstetric care. For example, of the mothers who gave birth in Finland in 2021, 18.4% were living with obesity at the start of their pregnancy [1]. Maternal pre-pregnancy obesity is a major risk factor for gestational diabetes mellitus (GDM), gestational hypertension and preeclampsia. It also increases the risk of Caesarian delivery, preterm birth (< 37 weeks of gestation), macrosomia, and admission to neonatal intensive care unit (NICU) [2–4]. Growing evidence suggests that maternal pre-pregnancy obesity also compromises the neurodevelopment of the children later in life [5–7]. While a substantial proportion of the mother–child adversities is attributable to maternal pre-pregnancy obesity [8], obesity alone is insufficient for the identification of all at-risk mothers and children, as many women with non-obesity (< 30 kg/m^2^) or normal weight (18.5–24.99 kg/m^2^) and their children may suffer from adversities [9]. Therefore, it is very important to identify biomarkers, which would perform better as predictors of the mother–child adverse outcomes than maternal BMI and which could be malleable targets of interventions aimed at reducing the risks for both the mothers and their children.

Maternal BMI-related metabolome may bear relevance in this context. Ample evidence shows that maternal pre-pregnancy obesity and/or higher BMI are associated with vast perturbations across multiple metabolic pathways during pregnancy, including lipoproteins and cholesterol [10–14], glycerides, phospholipids, amino acids (AA) [10–12, 14], fatty acids (FA) [10, 12–14], glycolysis [10, 12], ketone bodies [10, 12, 13], fluid balance, inflammation [10, 12, 14], carnitine, and measures related to the Krebs cycle, and oxidative stress [14]. Evidence is emerging that maternal BMI-related metabolic perturbations may improve the prediction of mother–child outcomes over maternal BMI. By using penalized lasso regression, one study among 682 mother–child dyads identified 43 maternal metabolic measures during pregnancy, which were associated with higher maternal pre-pregnancy BMI [14]. Of these, 19 metabolic measures, including AA, FA and glycerides, which were also associated with the child’s higher birth weight, improved the prediction of child’s birth weight over a model which included maternal pre-pregnancy BMI alone, or BMI in combination with conventional biomarkers, including maternal glucose, triglycerides and HDL-cholesterol [14]. Another study of 400 mother–child dyads identified maternal BMI-associated metabolic measures during pregnancy using per-metabolite linear models. In random forest analyses, the prediction of birth weight was improved by 15 BMI-related metabolic measures including acylcarnitines, lipids, triacylglycerols, AA, and mixed metabolites over a model which included maternal BMI, glucose, and child’s gestational age at birth [15]. Finally, in a study among 8,212 mother–child dyads, which used penalized elastic net regression, 33 to 147 maternal metabolic measures during pregnancy, including lipoproteins, cholesterol and monounsaturated FAs, improved the prediction of maternal hypertensive disorders, GDM and child’s small- (SGA) and large-for-gestational-age (LGA) birth weight over a model which included maternal pre-pregnancy BMI, age, parity, ethnicity and smoking during pregnancy [16]. However, none of these studies has used multivariate supervised analytical methods, allowing to cluster predictive information into one metabolomic component representing variation in the metabolic measures specifically explaining variation in maternal pre-pregnancy BMI. Such multivariate methods would allow accounting for the correlated nature of the metabolomics data and identifying variation in the metabolic measures that specifically reflect variation in maternal pre-pregnancy BMI. *Hence, while the findings from a few previous studies suggest that metabolic changes associated with maternal pre-pregnancy BMI may predict BMI-associated mother–child outcomes better than anthropometric BMI, further studies are warranted.*

We have previously identified maternal pre-pregnancy BMI-related metabolomic component based on 68 metabolomic measures by using Orthogonal Partial Least Squares (O-PLS) regression, which improved the prediction of any mental and behavioral disorder in the children over a model which included maternal pre-pregnancy BMI alone [17]. Here we extend these analyses by using the most recent quantification library for the metabolic measures [18], resulting in a metabolomic component based on 95 metabolic measures reflecting variation specific to maternal pre-pregnancy BMI, or, as it has been referred to by previous studies, BMI-defined metabolome [19]. We examined whether this maternal BMI-defined metabolome was associated with maternal GDM and hypertensive disorders during pregnancy, inadequate and excessive gestational weight gain (GWG), Caesarian delivery, child’s gestational age and weight at birth, preterm birth, admission to NICU, and neurodevelopment in childhood. We further tested whether the BMI-defined metabolome improved the prediction of these mother–child outcomes over maternal pre-pregnancy BMI. Finally, to facilitate replication, support the clinical utility of the findings, and test whether metabolic changes associated with higher BMI would predict adverse mother–child outcomes in other pregnant populations, and even at the level of non-obesity and normal weight, we developed novel polymetabolic risk scores (PMRSs) of high maternal pre-pregnancy BMI, and tested their performance in predicting the same set of mother–child outcomes in an independent cohort of 489 mother–child dyads.

## Materials and methods

### Participants

We enrolled 1079 pregnant mothers and their singleton children born alive between 2006–2010 to the Prediction and Prevention of Pre-eclampsia and Intrauterine Growth Restriction (PREDO) study [20]. The mothers had a known clinical risk-factor status for pre-eclampsia and intrauterine growth restriction: 969 had one or more and 110 had none of the clinical risk factors. Mothers were recruited from 10 study hospitals in Southern and eastern Finland when they arrived for their first ultrasound screening at 12–14 weeks of gestation. Inclusion and exclusion criteria for the PREDO study are provided in the Supplemental Table 1.

Of the 1079 pregnant mothers, 425 donated blood samples up to three times during pregnancy. Economic constraints restricted the blood sampling to the three largest study hospitals. Blood samples were taken at a median 13.0 (interquartile range (IQR) 12.6–13.4), 19.3 (IQR 19.0–19.7), and 27.0 (IQR 26.6–27.6) weeks of gestation. Of the 425 mothers, 354 (83.8%) provided blood samples at all three time points, 52 (14.6%) at two time points, and 10 (2.4%) at one time point. Of these women, 100% had data on pregnancy complications, birth outcomes, and child psychological development disorder diagnoses in a follow-up from birth until the median age of 10.4 (IRQ 9.4–11.3) years. These data were enriched by mother-reports on child developmental milestones available for 230 (54.1% of the 425 mother–child dyads with blood samples and child diagnoses data) children at the median age of 3.7 (IRQ 2.8–4.4) years.

In comparison to the PREDO mothers who did not donate blood, those in the subsample who did were younger (age at delivery 32.5 vs. 33.6 years; *p* = 0.001). There were no significant differences in the other study variables (*p* > 0.07).

Mother child-dyads for the independent replication cohort came from the prospective InTraUterine sampling in early pregnancy (ITU) study [21]. ITU enrolled 943 pregnant mothers and their singleton children born alive between 2012–2017. The mothers were recruited from two study hospitals in Southern Finland when they underwent the national voluntary fetal 21-trisomy screening in early pregnancy. Of them, 399 had a negative and 544 had a positive screening result. Those with a positive screening result underwent fetal chromosomal testing but were cleared from fetal chromosomal abnormalities. Inclusion and exclusion criteria for the ITU study are provided in the Supplemental Table 1.

Of the 943 mothers, 489 donated one blood sample at a median 20.6 (IRQ 20.1–23.7) weeks of gestation. Of the 489 mothers who donated blood, 100% had data on pregnancy complications, birth outcomes and child psychological development disorder diagnoses in a follow-up from birth until the median age of 4.6 (IRQ 3.8–5.3) years. These data were enriched by mother reports of child developmental milestones available for 376 (76.9% of the 489 mother–child dyads with blood samples and child diagnoses data) children at the median age of 1.5 (IRQ 1.5–1.6) years, and by neurocognitive test results available for 400 (81.8%) of the children at the median age of 2.9 (IRQ 2.8–3.0) years.

In comparison to the ITU mothers who did not donate blood, those in the subsample who did more often had tertiary education (83.7% vs. 70.0%), had lower BMI (23.7 vs. 24.6 kg/m^2^; *p* = 0.004), and smoked less often during pregnancy (2.4% vs. 5.0%, *p* = 0.001), but there were no significant differences in the other study variables (*p* > 0.20).

The PREDO and ITU study protocols were approved by the ethics committees of the Helsinki and Uusimaa Hospital District and are in alignment with the Code of Ethics of the World Medical Association (Declaration of Helsinki). All participants and guardian(s) of minors provided written informed consent forms. The consents enabled linkage to nationwide medical register data for the mothers and the children using unique personal identification numbers assigned to all Finnish citizens and residents since 1969.

### Maternal pre-pregnancy BMI

In the PREDO and ITU cohorts, maternal pre-pregnancy BMI was extracted from the Medical Birth Register (MBR). Pre-pregnancy BMI was calculated from weight and height verified at the first visit to the antenatal clinic between 7–10 weeks of gestation.

### Maternal metabolic measures during pregnancy

Venous blood samples were drawn from the antecubital vein between 7 and 10 AM after at least a 10-h overnight fast in PREDO and between 7 and 9 AM after a 12-h overnight fast in ITU. Plasma was separated immediately and stored at − 80 °C until analysis. A high-throughput proton nuclear magnetic resonance (NMR) metabolomics platform quantified 225 metabolic measures using the Nightingale Health Quantification Library 2020 [18] (Nightingale Health Ltd, Helsinki, Finland) (https://nightingalehealth.com/about/technology). Following the lead of earlier studies using this NMR metabolomics platform, in our analyses, we used 95 metabolic measures covering multiple metabolic pathways, including lipoproteins and cholesterol, glycerides, phospholipids, AA, FA, glycolysis, ketone bodies, fluid balance, and inflammation [22, 23]. Details of the experimentation and applications of the NMR metabolomics platform have been described previously [24]. Of the metabolic measures, 39 including cholesterols, triglycerides, apolipoproteins, FA, AA, glycolysis related metabolites, fluid balance and inflammation have been validated against clinical chemistry methods (https://research.nightingalehealth.com/clinically-validated-biomarkers).

### Pregnancy complications

In PREDO and ITU, data on maternal gestational diabetes mellitus (GDM) and hypertensive disorders during pregnancy were extracted from the MBR, and/or the Finnish Care Register for Health Care (HILMO). In the PREDO, the diagnoses of hypertensive disorders were further verified from the medical records by an expert jury comprising two medical doctors and a research nurse with expertise in obstetrics and gynecology.

According to the Finnish clinical care guidelines, GDM is defined as fasting, 1 or 2 h plasma glucose during a 75 g oral glucose tolerance test ≥ 5.3, 10.0, or 8.6 mmol/L that emerged or was first identified during pregnancy (coded using the International Statistical Classification of Diseases and Health-Related Problems 10th Revision [ICD-10] O24.4); Gestational hypertension as blood pressure ≥ 140/90 mmHg on ≥ 2 occasions at least 4 h apart in a woman who was normotensive before 20 weeks of gestation (ICD-10: O13); Pre-eclampsia as blood pressure ≥ 140/90 mmHg on ≥ 2 occasions at least 4 h apart in a woman who was normotensive before 20 weeks of gestation with proteinuria ≥ 300 mg/24 h (ICD-10: O11, O14, O15); Chronic hypertension blood pressure ≥ 140/90 mmHg SBP/DBP present from pre-pregnancy or before 20 weeks of gestation onwards (ICD-10: O10, I10). In the analyses of GDM, normoglycemic women were used as the comparison, and in the analyses of hypertensive disorders, the comparison group comprised normotensive women.

GWG was calculated as a difference between the weight verified at the first antenatal visit and weight measured at delivery. In both cohorts, weight at the first antenatl visit was extracted from the MBR, and weight at delivery from medical records. To classify inadequate, normal and excess GWG during pregnancy in different pre-pregnancy BMI categories we used the Institute of Medicine guidelines (normal GWG in women with: underweight 12.5–18 kg; normal weight: 11.5–16.0 kg; overweight: 7.0–11.5 kg; obesity: 5.0–9.0 kg) [25]. GWG below and above normal GWG is vategorized as inadequate and excess, respectively.

### Birth outcomes

In both cohorts, data on the mode of delivery (Caesarean vs vaginal), child’s gestational age (preterm < 37 weeks of gestation vs term ≥ 37 weeks of gestation) and birth weight (g) and NICU admission (yes vs no) were extracted from the medical records and/or the MBR.

### Child neurodevelopmental outcomes

In PREDO, psychological development disorders were identified from HILMO from birth until 31 December 2018, when the children were 8.4–12.8 years old. In ITU, these disorders were identified from HILMO from birth until 31 December 2020 when the children were 3.1–8.7 years old. HILMO includes all in-patient hospitalizations (since 1969) in Finland and all outpatient treatments (since 1998) by physicians in public specialized-care, and covers psychiatric diagnoses well [26]. Psychological development disorders included the following diagnoses: developmental disorders of speech and language (ICD-10: F80), scholastic skills (F81) and motor function (F82), mixed specific developmental disorders (F83), and autism spectrum disorders (F84).

In addition to the diagnoses, neurodevelopment of the children in both cohorts was assessed using the age-appropriate Ages and Stages Questionnaires (ASQ) [27]. The ASQ was completed by the child’s mother and comprises 30 items measuring skills appropriate for the child’s age in the domains of communication, fine and gross motor function, problem solving, and personal/social skills. A score that is at or below -2SDs of the age-appropriate mean is considered to represent neurodevelopmental delay [27].

Using information from both the HILMO and the ASQ, and to increase statistical power, we created one broad outcome variable based on the number of areas in which the child displayed neurodevelopmental delay: (1) delay in cognitive development (F80, F81, F83 or scoring ≤ -2SD for age on ASQ communication and/or problem solving skills; (2) delay in motor development (F82 or scoring ≤ -2SD for age on ASQ fine motor and/or gross motor skills), (3) delay in social development (F84 or scoring ≤ -2SD for age on ASQ personal/social skills). Hence, this variable has three levels and captures delay in all three (3), delay in any two (2), delay in any one (1) and no delay in any area (0) [28].

In the ITU cohort, we additionally assessed child neurodevelopment with Bayley Scales of Infant and Toddler Development, Third Edition (Bayley III) [29]. Bayley-III is a comprehensive tool assessing cognitive, language and motor development in children from 16 days to 42 months of age [30]. Bayley-III is a tool with high concurrent validity [31] and is widely used in clinical practice to evaluate neurodevelopment in young children [30]. Trained master’s students of psychology administered the test under the supervision of a clinical neuropsychologist (EW).

### Covariates

The models predicting maternal pregnancy complications and birth outcomes were adjusted for maternal age (years) and parity (primiparous vs multiparous) derived from MBR and/or HILMO, maternal education level (secondary or lower vs tertiary) self-reported in early pregnancy and maternal substance use (yes vs no) combining data on smoking during pregnancy derived from MBR and self-reported data on alcohol consumption in early pregnancy [10, 28]. The models predicting birth weight additionally included gestational age at birth and sex of the child, and the models predicting neurodevelopmental delay and Bailey-III scales additionally included child’s sex and birth year or age at testing which we derived from MBR, HILMO, or clinical visit date. We did not include maternal hypertensive and diabetes disorders, GWG, birth weight or gestational age in the models predicting child neurodevelopmental outcomes, as they may lie on the same pathway with maternal pre-pregnancy BMI, and hence mediate rather than confound the potential associations with neurodevelopmental outcomes.

### Statistical analyses

#### Identification of maternal BMI-defined metabolome

In the PREDO cohort, we identified maternal BMI-defined metabolome using the O-PLS regression. O-PLS is a multivariate statistical technique used for modeling the relationship between a set of predictor variables and a response variable. The model is decomposed into predictive and orthogonal components. The predictive component is a continuous variable capturing the variation in the predictor variables that explains the variation in the response variable [32]. We regressed maternal pre-pregnancy BMI on the 95 metabolic measures, which we averaged across the three consecutive time-points, as the metabolic measures showed high intra-class correlations over time (*r* = 0.56–0.89, *p* < 0.001), and as our previous analysis showed that obese pregnancies were characterized by persistent metabolic perturbations throughout pregnancy and smaller change across the three measurement points [10]. The predictive component representing variation in the metabolic measures capturing variation in maternal pre-pregnancy BMI extracted from O-PLS model represented BMI-defined metabolome. We used it as a predictor of pregnancy complications, birth outcomes and child neurodevelopment in PREDO. Before applying the O-PLS regression, metabolic measures below the detection level were replaced with a value of 0.9 multiplied by the minimum value of that measurement [33]. All metabolic measures were log-transformed and standardized to the mean of 0 and standard deviation (SD) of 1. All values above and below 5 SD from the mean were considered outliers and recoded as missing values [10].

#### Generation of the high maternal BMI-related PMRSs

Based on the BMI-defined metabolome identified in the PREDO cohort, in the independent ITU cohort, we generated aggregate PMRSs of weighted metabolic measures, which in the PREDO were associated with maternal pre-pregnancy BMI. PMRSs were calculated using different variable importance for the projection (VIP) thresholds (> 0.7, > 1.0, > 1.2, > 1.4), denoting contributions by each metabolic measure to the variance in BMI-defined metabolome. We then selected the metabolic measures in the ITU cohort up to the varying VIP thresholds, weighted them by the loadings on the maternal pre-pregnancy BMI predictive component derived from the O-PLS regression in the PREDO cohort (Supplemental Table 2) and summed them up to obtain the PMRSs. Before weighting the metabolic measures, we re-coded each metabolic measure into -1, 0 and 1, if they fell below the 5th, between the 5th and 95th, and above the 95th percentile of the value of each metabolic measure in normal weight, normoglycemic and normotensive women in PREDO (Supplemental Table 3). Using the values of normal weight, normoglycemic and normotensive women in PREDO cohort as referents allows for calculating the PMRSs and replicating our findings in other cohorts. We standardized the PMRSs to the mean of 0 and SD of 1 and studied their performance in predicting pregnancy complications, birth outcomes and child neurodevelopment in ITU. Supplemental material 1 contains a detailed description of PMRS generaion as well as a SAS code that we have used to generate PMRS. The PMRS generated in this study can be applied to any other sample that utilizes the NMR platform.

#### Associations between maternal BMI-defined metabolome and BMI-related PMRSs with pregnancy complications, birth outcomes and child neurodevelopment

To examine associations between maternal BMI-defined metabolome in PREDO and the PMRSs of high pre-pregnancy BMI in ITU with pregnancy complications, birth outcomes and child neurodevelopment, we used logistic (binary outcomes), linear (continuous outcomes) and Poisson regression (outcomes with count data). We first conducted these analyses in all mothers and children in the two cohorts. To examine whether the PMRSs would allow identification of at-risk women and children also among mothers with non-obesity and normal weight mothers, we restricted the analyses in ITU to non-obese and normal weight groups. Finally, to examine whether maternal BMI-defined metabolome and PMRSs of high maternal BMI improved the prediction of the mother–child outcomes over maternal pre-pregnancy anthropometric BMI, we tested the goodness-of-fit of two nested models. We compared the fit of a baseline model, in which maternal pre-pregnancy BMI was a sole predictor of the mother–child outcomes, with the fit of a model, which included both maternal pre-pregnancy BMI and BMI-defined metabolome in PREDO and maternal pre-pregnancy BMI and PMRSs (each PMRS tested in a separate model) in ITU as the predictors. The likelihood ratio chi-square test (LRT) assessed whether the BMI-defined metabolome and the PMRS improved the prediction of the mother–child outcomes over maternal pre-pregnancy BMI. We also calculated the change in the amount of variation explained by the maternal BMI-defined metabolome/PMRS over maternal pre-pregnancy BMI by examining changes in Nagelkerke Pseudo R2/ R2 values between the two nested models. As effect size estimates we present mean differences (MD), Odds Ratios (OR) and Relative Risks (RR) and their 95% Confidence Intervals (CI).

O-PLS regression analyses were performed using SIMCA (Version 17.0, Umetrics, Sweden). All other statistical analyses were performed using SAS 9.4 (SAS Institute Inc., Cary, NC, USA).

## Results

Descriptive characteristics of the PREDO and ITU cohorts are shown in Table 1. In the ITU cohort, the mothers were older, more educated, had lower pre-pregnancy BMI and less often had hypertensive disorders in comparison to the mothers in the PREDO cohort (all p-values < 0.0001). Children in the ITU cohort had higher gestational ages at birth (*p* < 0.0001) and were less likely to be admitted to NICU (*p* = 0.003) in comparison to the children in the PREDO cohort.

**Table 1:** Descriptive statistics of the study population (PREDO) and replication cohort (ITU)

	**PREDO (*N* = 425 mother–child dyads)**	**ITU (*N* = 489 mother–child dyads)**	
	**Mean (SD) or N (%)**	**Mean (SD) or N (%)**	**p**
**Maternal characteristics**			
Maternal age, years	32.5 (5.3)	34.9 (4.7)	< 0.0001
Education			< 0.0001
Primary or secondary education	204 (49.0%)	78 (16.3%)	
University degree	212 (51.0%)	400 (83.7%)	
Data not available	9 (2.1%)	11 (2.3%)	
Maternal BMI, kg/m^2^	27.0 (6.5)	23.7 (3.9)	< 0.0001
Gestational diabetes during pregnancy			
No gestational diabetes	335 (78.8%)	392 (80.2%)	0.62
Gestational diabetes	90 (21.2%)	97 (19.8%)	
Hypertensive disorders during pregnancy			< 0.0001
Normotension	271 (63.8%)	461 (94.3%)	
Gestational hypertension	39 (9.2%)	7 (1.4%)	
Preeclampsia	43 (10.1%)	15 (3.1%)	
Chronic hypertension	72 (17.0%)	6 (1.2%)	
Gestational weight gain			0.07
Inadequate	57 (22.5%)	117 (24.1%)	
Normal	77 (30.3%)	181 (37.2%)	
Excessive	119 (47.0%)	188 (38.7%)	
Data not available	172 (40.5%)	2 (0.4%)	
**Birth outcomes**			
Child sex			0.52
Boy	228 (53.7%)	252 (51.5%)	
Girl	197 (46.4%)	237 (48.5%)	
Delivery mode			0.99
Vaginal delivery	324 (78.8%)	386 (78.0%)	
Caesarian section	87 (21.2%)	103 (21.1%)	
Data not available	14 (3.3%)		
Gestational age, weeks	39.5 (2.0)	40.0 (1.7)	< 0.0001
Term birth	394 (92.7%)	468 (95.7%)	0.06
Preterm birth	31 (7.3%)	21 (4.3%)	
Birth weight, grams	3464.1 (610.6)	3505.6 (499.1)	0.05
Admission to neonatal intensive care unit			0.003
No	346 (83.0%)	439 (89.8%)	
Yes	71 (17.0%)	50 (10.2%)	
Data not available	8 (1.9%)		
**Neurodevelopment**			
Cognitive developmental delay (F80, F81, F83 or ≤ − 2 SD ASQ communication or problem-solving skills)a	36 (8.5%)	38 (7.8%)	0.70
Motor developmental delay (F82 or ≤ − 2 SD ASQ fine or gross motor skills)a	26 (6.1%)	25 (5.1%)	0.51
Social developmental delay (F84 or ≤ − 2 SD ASQ personal social skills)a	13 (3.1%)	17 (3.5%)	0.73
Number of neurodevelopmental delay across the 3 broad areas			0.10
No delay in any area	370 (88.5%)	424 (86.7%)	
Delay in any of the areas	29 (6.9%)	51 (10.4%)	
Delay in any 2 of the areas	15 (3.6%)	13 (2.7%)	
Delay in all three areas	4 (1.0%)	1 (0.2%)	
Mean age at neurodevelopmental delay assessment, years	9.9 (2.4)	4.7 (1.4)	< 0.0001
Bayley Scales of Infant and Toddler Development			
Receptive language development		39.3 (2.8)	
Data not available		140 (28.6%)	
Expressive language development		42.1 (3.7)	
Data not available		132 (27.0%)	
Fine motor development		50.8 (4.5)	
Data not available		135 (27.6%)	
Gross motor development		63.6 (2.6)	
Data not available		283 (57.9%)	
Cognitive development		76.6 (3.4)	
Data not available		104 (21.2%)	
Mean age at Bayley Scales assessment, years		3.0 (0.2)	
Data not available		98 (20.%)	

There is no missing data unless indicated otherwise

### Identification of maternal BMI-defined metabolome and its associations with pregnancy complications, birth outcomes and child neurodevelopment

The O-PLS model regressing maternal pre-pregnancy BMI on 95 metabolic measures demonstrated a good model fit explaining 58.4% of the total variation in maternal pre-pregnancy BMI with a predictive ability of 52.4%. Loadings of the 95 metabolic measures on maternal BMI-defined metabolome are shown in Supplemental Figs. 1 and 2 shows loadings of the 95 metabolic measures at each of the blood sampling points on maternal BMI-defined metabolome demonstrating that maternal pre-pregancy BMI-defined metabolome remained quite consistent across pregnancy. Metabolites with the highest positive loadings represented inflammation, monounsaturated FA, glycolysis, ketone bodies and lipids, and metabolites with the highest negative loadings represented FA composition, fluid balance and AA. Metabolic measures contributing to maternal BMI-defined metabolome and their corresponding VIPs are shown in Supplemental Fig. 3. Histogram showing the distributions of maternal BMI-defined metabolome in women with normal weight, overweight and obesity is shown in Supplemental Fig. 4.

Maternal BMI-defined metabolome was associated with significantly higher ORs of GDM, gestational and chronic hypertension, preeclampsia, inadequate and excessive GWG, Caesarean section, with higher child birth weight and lower child gestational age, and with higher OR of preterm birth and admission to NICU (Table 2). Maternal BMI-defined metabolome was also associated with a higher number of areas, in which the child displayed neurodevelopmental delay (Table 2). In mothers with non-obesity maternal BMI-defined metabolome was associated with all mother–child outcomes except for inadequate and excessive GWG and Caesarian section delivery (Table 3) and in mothers with normal weight maternal BMI-defined metabolome was associated with GDM, chronic hypertension, admission to NICU and higher number of neurodevelopmental delay areas (Supplemental Table 4).

**Table 2:** Associations of maternal BMI-defined metabolome and BMI-related PMRSs with maternal pregnancy complications, birth outcomes and child neurodevelopment

	PREDO, BMI-defined metabolome, SD units			ITU, BMI-related PMRS VIP^a^ 0.7 SD units			ITU, BMI-related PMRS VIP 1.0 SD units			ITU, BMI-related PMRS VIP 1.2 SD units			ITU, BMI-related PMRS VIP 1.4 SD units		
	OR/MD	95% CI	p	OR/MD	95% CI	p	OR/MD	95% CI	p	OR/MD	95% CI	p	OR/MD	95% CI	p
**Maternal pregnancy compications**															
GDM, yes vs. no	2.52	1.92, 3.31	< 0.0001	1.61	1.28, 2.03	< 0.0001	1.63	1.29, 2.06	< 0.0001	1.66	1.31, 2.09	< 0.0001	1.66	1.31, 2.09	< 0.0001
Hypertensive pregnancy disorders															
Normotension	Ref			Ref			Ref			Ref			Ref		
Gestational hypertension, yes vs. no	1.46	1.00, 2.12	0.05	1.89	0.91, 3.92	0.09	1.83	0.88, 3.81	0.11	1.88	0.90, 3.91	0.09	2.01	0.97, 4.14	0.06
Preeclampsia, yes vs. no	2.24	1.56, 3.24	< 0.0001	2.04	1.18, 3.52	0.01	2.08	1.20, 3.61	0.009	1.94	1.13, 3.34	0.02	1.92	1.12, 3.29	0.02
Chronic hypertension, yes vs. no	2.26	1.66, 3.07	< 0.0001	1.49	0.60, 3.73	0.39	1.47	0.59, 3.68	0.41	1.46	0.59, 3.63	0.42	1.65	0.66, 4.09	0.28
Gestational weight gain															
Normal	Ref			Ref			Ref			Ref			Ref		
Inadequate, yes vs. no	1.72	1.15, 2.58	0.008	0.90	0.70, 1.16	0.41	0.92	0.72, 1.18	0.53	0.93	0.72, 1.19	0.54	0.93	0.73, 1.19	0.57
Exessive, yes vs. no	1.63	1.14, 2.35	0.008	1.05	0.84, 1.30	0.68	1.05	0.85, 1.31	0.63	1.02	0.82, 1.27	0.83	1.04	0.84, 1.29	0.74
**Birth outcomes**															
Caesarian section vs. vaginal delivery	1.28	1.00, 1.64	0.05	1.28	1.02, 1.61	0.03	1.29	1.02, 1.62	0.03	1.31	1.04, 1.65	0.02	1.33	1.06, 1.67	0.02
Gestational age, weeks	-0.30	-0.50, -0.09	0.005	-0.15	-0.30, 0.00	0.06	-0.15	-0.30, 0.00	0.05	-0.14	-0.29, 0.01	0.06	-0.14	-0.29, 0.01	0.07
Preterm birth, yes vs. no	1.54	1.06, 2.23	0.02	1.15	0.74, 1.78	0.54	1.13	0.73, 1.75	0.60	1.08	0.70, 1.68	0.73	1.15	0.74, 1.78	0.53
NICU treatment, yes vs. no	1.63	1.25, 2.14	0.0003	1.42	1.05, 1.92	0.02	1.39	1.03, 1.88	0.03	1.37	1.01, 1.85	0.04	1.41	1.05, 1.91	0.02
Birth weight, grams	91.84	46.59, 137.08	< 0.0001	34.48	-2.40, 71.35	0.07	33.47	-3.43, 70.37	0.08	27.67	-9.41, 64.75	0.14	19.08	-18.00, 56.13	0.31
**Child neurodevelopment**															
Number of areas of child neurodevelopmental delay^b^	1.48	1.18, 1.87	0.0008	1.00	0.79, 1.25	0.97	0.99	0.70, 1.25	0.95	1.00	0.79, 1.25	0.97	0.98	0.78, 1.24	0.88
Bayley Scales of Infant and Toddler Development, raw units															
Receptive language development				-0.03	-0.30, 0.24	0.81	-0.04	-0.30, 0.23	0.78	-0.06	-0.32, 0.21	0.67	-0.05	-0.31, 0.22	0.73
Expressive language development				0.05	-0.31, 0.41	0.77	0.02	-0.34, 0.38	0.93	0.06	-0.30, 0.42	0.75	0.07	-0.29, 0.43	0.72
Fine motor development				0.20	-0.14, 0.54	0.25	0.19	-0.14, 0.53	0.26	0.18	-0.15, 0.52	0.29	0.21	-0.13, 0.55	0.23
Gross motor development				-0.09	-0.38, 0.20	0.54	-0.11	-0.40, 0.17	0.43	-0.14	-0.43, 0.16	0.36	-0.13	-0.43, 0.17	0.38
Cognitive development				-0.30	-0.58, -0.01	0.04	-0.30	-0.58, -0.01	0.04	-0.30	-0.59, -0.01	0.04	-0.24	-0.53, 0.05	0.10

^a^*VIP* Variable importance for projection

^b^Measure of association is relative risk (RR)

**Table 3:** Associations of maternal BMI-defined metabolome and BMI-related PMRSs with maternal pregnancy complications, birth outcomes and child neurodevelopment in the subsample of women with nonobesity

	PREDO, BMI-defined metabolome, SD units			ITU, BMI-related PMRS VIP^a^ 0.7 SD units			ITU, BMI-related PMRS VIP 1.0 SD units			ITU, BMI-related PMRS VIP 1.2 SD units			ITU, BMI-related PMRS VIP 1.4 SD units		
	OR/MD	95% CI	p	OR/MD	95% CI	p	OR/MD	95% CI	p	OR/MD	95% CI	p	OR/MD	95% CI	p
**Maternal pregnancy compications**															
GDM, yes vs. no	2.33	1.49, 3.64	0.0002	1.65	1.27, 2.12	0.0001	1.70	1.29, 2.16	< 0.0001	1.68	1.30, 2.18	< 0.0001	1.65	1.27, 2.14	0.0001
Hypertensive pregnancy disorders															
Normotension	Ref			Ref			Ref			Ref			Ref		
Gestational hypertension, yes vs. no	1.96	1.05, 3.68	0.04	1.56	0.70, 3.48	0.28	1.51	0.68, 3.36	0.32	1.57	0.70, 3.49	0.27	1.72	0.78, 3.78	0.18
Preeclampsia, yes vs. no	3.34	1.73, 6.43	0.0003	1.99	1.13, 3.50	0.02	1.99	1.13, 3.51	0.02	1.85	1.05, 3.25	0.03	1.88	1.07, 3.27	0.03
Chronic hypertension, yes vs. no	2.65	1.50, 4.77	0.001	0.48	0.09, 2.64	0.40	0.48	0.09, 2.48	0.38	0.44	0.09, 2.17	0.32	0.44	0.09, 2.18	0.32
Gestational weight gain category															
Normal	Ref			Ref			Ref			Ref			Ref		
Inadequate, yes vs. no	1.40	0.82, 2.40	0.21	0.88	0.68, 1.14	0.33	0.90	0.70, 1.16	0.42	0.90	0.69, 1.16	0.72	0.90	0.70, 1.16	0.41
Exessive, yes vs. no	1.11	0.66, 1.87	0.68	1.04	0.83, 1.31	0.68	1.05	0.84, 1.32	0.66	1.02	0.82, 1.27	0.83	1.03	0.82, 1.30	0.78
**Birth outcomes**															
Caesarian section vs. vaginal delivery	1.35	0.89, 2.05	0.16	1.16	0.91, 1.48	0.22	1.17	0.91, 1.49	0.22	1.19	0.94, 1.53	0.15	1.20	0.94, 1.53	0.14
Gestational age, weeks	-0.64	-0.97, -0.32	0.0001	-0.07	-0.21, 0.08	0.37	-0.07	-0.21, 0.08	0.37	-0.07	-0.22, 0.07	0.32	-0.08	-0.22, 0.07	0.30
Preterm birth, yes vs. no	2.21	1.22, 4.02	0.009	1.05	0.65, 1.73	0.82	1.01	0.62, 1.66	0.97	0.98	0.60, 1.61	0.94	1.05	0.62, 1.72	0.84
NICU treatment, yes vs. no	1.75	1.11, 2.74	0.02	1.29	0.94, 1.78	0.12	1.26	0.91, 1.73	0.17	1.24	0.90, 1.72	0.18	1.30	0.94, 1.79	0.11
Birth weight, grams	75.66	8.23, 143.09	0.03	21.94	-15.25, 59.14	0.25	22.93	-14.28, 60.14	0.23	17.98	-19.51, 55.47	0.34	8.64	-28.82, 46.10	0.65
**Neurodevelopment**															
Number of areas of child neurodevelopmental delay^b^	2.28	1.52, 3.41	< 0.0001	1.08	0.85, 1.37	0.54	1.07	0.84, 1.35	0.59	1.07	0.84, 1.36	0.57	1.06	0.84, 1.35	0.63
Bayley Scales of Infant and Toddler Development, raw units															
Language development				0.09	-0.21, 0.40	0.54	0.09	-0.22, 0.39	0.58	0.08	-0.23, 0.39	0.6	0.07	-0.23, 0.38	0.64
Motor development				0.16	-0.24, 0.56	0.43	0.14	-0.26, 0.54	0.49	0.10	-0.30, 0.50	0.62	0.15	-0.25, 0.55	0.46
Cognitive development				-0.13	-0.29, 0.03	0.11	-0.13	-0.29, 0.03	0.10	-0.15	-0.31, 0.01	0.07	-0.13	-0.29, 0.03	0.10

^a^*VIP* Variable importance for projection

^b^Measure of association is relative risk (RR)

**Fig. 1:**
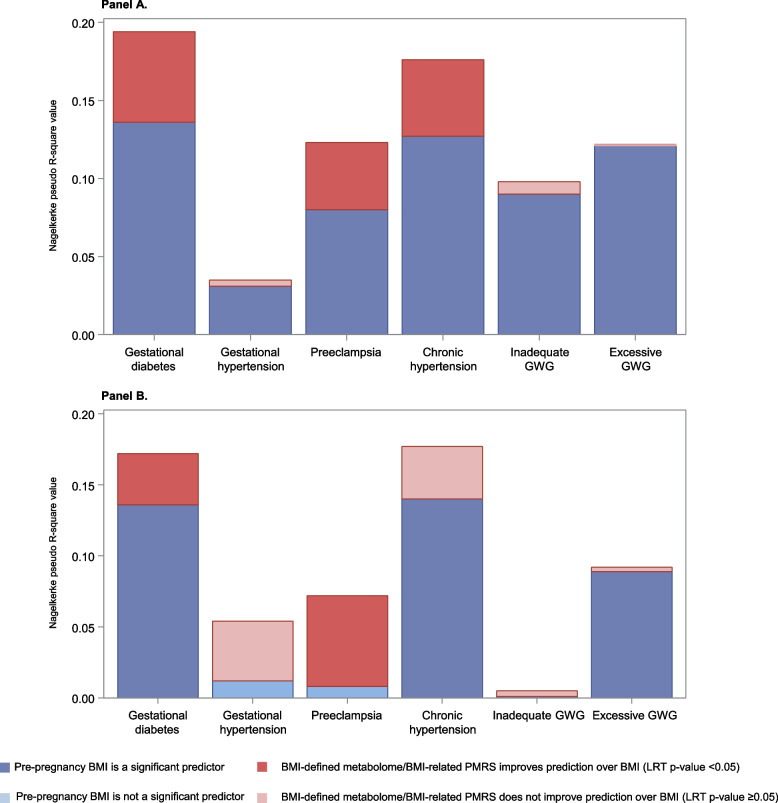
**A** Improvement of prediction of pregnancy complications by maternal BMI-defined metabolome over maternal pre-pregnancy BMI, PREDO. **B** Improvement of prediction of pregnancy complications by maternal pre-pregnancy BMI-related PMRS over maternal pre-pregnancy BMI, ITU

**Fig. 2:**
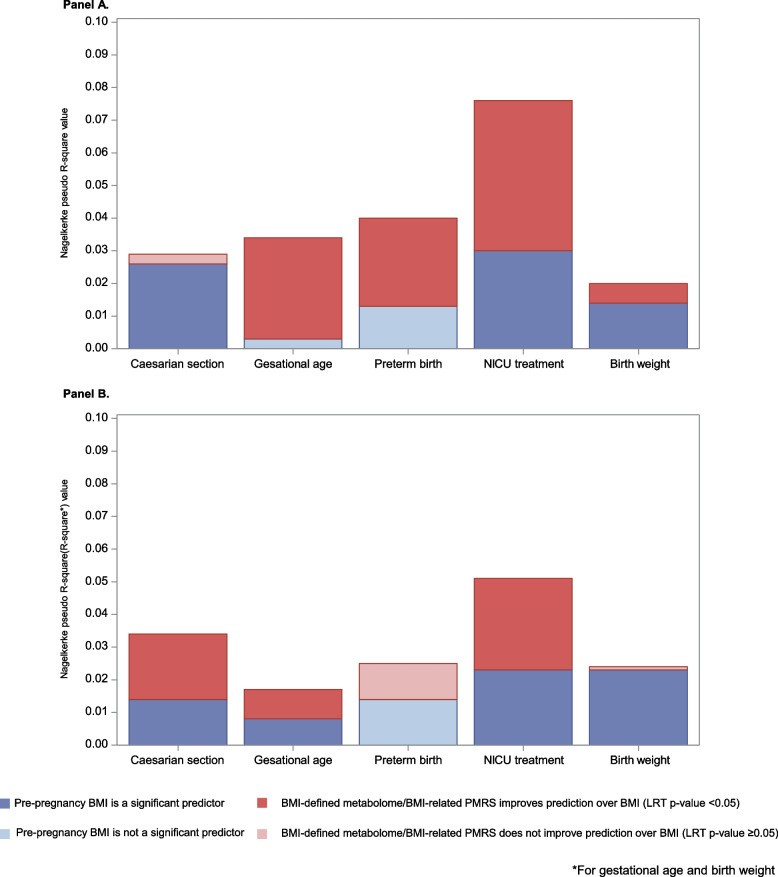
**A** Improvement of prediction of birth outcomes by maternal BMI-defined metabolome over maternal pre-pregnancy BMI, PREDO. **B** Improvement of prediction of birth outcomes by maternal pre-pregnancy BMI-related PMRS over maternal pre-pregnancy BMI, ITU

**Fig. 3:**
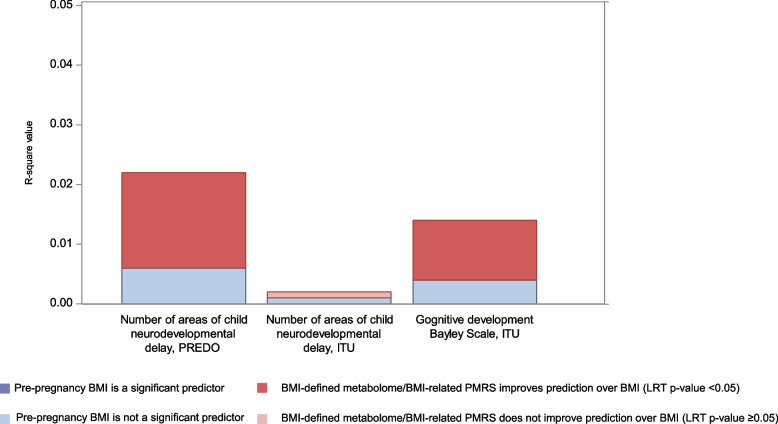
Improvement of prediction of child neurodevelopment by maternal BMI-defined metabolome / BMI-related PMRS over maternal pre-pregnancy BMI

In comparisons of the two nested models, maternal BMI-defined metabolome improved the prediction over maternal pre-pregnancy BMI as a sole predictor of GDM (5.8% increase in explained variance), preeclampsia (4.3% increase), chronic hypertension (4.9% increase) (Fig. 1A), gestational age (3.1% increase), birth weight (0.6% increase), preterm birth (2.7% increase), NICU admission (4.6% increase) (Fig. 2A), and the number of areas the child presented with neurodevelopmental delay (1.6% increase) (Fig. 3.).

### Generation of the high maternal pre-pregnancy BMI-related PMRSs and associations with pregnancy complications, birth outcomes and child neurodevelopment

PMRSs of high maternal pre-pregnancy BMI generated based on metabolic measures at different VIP thresholds (Supplemental Fig. 3) correlated with maternal BMI (*r* = 0.28–0.29, *p* < 0.0001) and with each other (*r* = 0.92–0.99, *p* < 0.0001). At the same time, PMRSs of high BMI identified women with non-obesity and normal weight women who displayed metabolic changes associated with high BMI. Histograms showing the distributions of the PMRSs in women with normal weight, overweight and obesity are shown in Supplemental Fig. 4.

All PMRSs were associated with significantly higher ORs of GDM, preeclampsia, Caesarean section and admission to NICU (Table 2). Additionally, PMRSs were marginally predictive of lower gestational age, and for PMRS VIP 1.2. this association was significant. While the PMRSs were not associated with child neurodevelopmental delay, all PMRSs but PMRS VIP 1.4. were associated with significantly lower child cognitive development score (Table 2).

In mothers with non-obesity, all PMRSs were associated with significantly higher ORs of GDM and preeclampsia (Table 3), and in the normal weight group, all PMRSs were associated with significantly higher ORs of GDM, preeclampsia, Caesarean section, NICU admission and with child’s lower gestational age at birth. For comparison, in mothers with non-obesity, maternal anthropometric BMI predicted only maternal GDM and child’s birth weight, and in mother with normal weight it only predicted lower gestational age at birth.

When we compared the two nested models, all PMRSs improved the prediction over maternal pre-pregnancy BMI as a sole predictor of GDM (3.1–3.6% increase in explained variance, best predictor PMRS VIP 1.2), preeclampsia (6.0–7.4% increase, best predictor PMRS VIP 0.7) (Fig. 1B), Caesarean Sect. (1.5–2.0% increase, best predictor PMRS VIP 1.2), gestational age (0.8–0.9% increase, best predictor PMRS VIP 1.2), NICU admission (2.5–2.8% increase, best predictor PMRS VIP 0.7) (Fig. 2B) and cognitive development score (0.9–1.0% increase, best predictor PMRS VIP 1.2) (Fig. 3.).

## Discussion

Our study demonstrated that metabolic changes characterizing high maternal pre-pregnancy BMI, referred to as the BMI-defined metabolome, were associated with maternal pregnancy complications, birth outcomes, and child neurodevelopment. Maternal BMI-defined metabolome was associated with higher odds of maternal GDM, chronic and gestational hypertension, preeclampsia, inadequate and excessive GWG, Caesarean section delivery, child’s admission to NICU and preterm birth. It was also associated with the child’s lower gestational age and higher birth weight, and with neurodevelopmental delay in childhood. Based on metabolic measures with the highest contributions to BMI-defined metabolome representing inflammation, FA composition, glycolysis, fluid balance, ketone bodies, lipids and AA, we created novel PMRSs of higher maternal pre-pregnancy BMI. In an independent replication cohort, the PMRSs were associated with higher odds of GDM, preeclampsia, Caesarean section and child’s admission to NICU, and poorer cognitive development score in childhood. The PMRSs were able to predict GDM, preeclampsia, Caesarean section, child’s admission to NICU, and lower gestational age even at the levels of maternal pre-pregnancy non-obesity and/or normal weight. Finally, this study showed that both maternal BMI-defined metabolome and PMRSs of high maternal BMI improved the prediction of most of the examined mother–child outcomes. Our findings thus suggest that metabolic perturbations associated with higher maternal pre-pregnancy BMI may offer insight into the underpinning biological mechanisms explaining associations of higher maternal pre-pregnancy BMI with adverse mother–child outcomes and show that the novel PMRSs perform better than maternal anthropometric pre-pregnancy BMI in identifying at-risk women and their children for timely targeted personalized prevention interventions.

Our findings are in agreement with the findings from previous studies showing that metabolic changes observed in pregnant women with higher BMI improved the prediction of GDM, hypertensive disorders during pregnancy [16] and higher birth weight [14, 15] over anthropometric BMI. At the same time, our study added novel information and contributed to the organization and systematization of previously gathered evidence, as we have examined associations with a wider range of mother–child outcomes associated with maternal pre-pregnancy BMI than any of the previously published studies.

High maternal pre-pregnancy BMI is associated with a number of pathophysiological processes manifested at the level of maternal metabolome during pregnancy [11]. In agreement with previous findings, metabolic perturbations associated with higher maternal pre-pregnancy BMI in our study reflect low-grade inflammation, dyslipidemia, oxidative stress and increased insulin resistance [11, 34, 35]. All these biological mechanisms have been suggested to underlie associations between maternal obesity and pregnancy complications, adverse birth outcomes and suboptimal neurodevelopment of the child [11, 34–38]. On the other hand, FA composition and AA levels, which, in agreement with previous findings, in our study were also perturbed by higher BMI, are influenced by maternal diet [35] providing opportunities for intervention. For example, diets with high levels of saturated FA modulate inflammatory processes, while consumption of monounsaturated FA and polyunsaturated FA has positive effects on glucose metabolism and reduction in the levels of low density and very low density lipoproteins (LDL and VLDL) [39]. Dietary supplementation with AA regulates antioxidative reactions and reduces insulin resistance [40]. Therefore, our findings contribute to the evidence suggesting that dietary-based interventions aimed at influencing biological mechanisms underlying maternal BMI-related metabolic perturbations may benefit the mother and the child.

Our findings showed that while the maternal BMI-defined metabolome, BMI-related PMRSs and maternal anthropometric BMI were correlated, the PMRSs were able to identify women who were metabolically unhealthier than their anthropometric BMI would indicate. This finding is also in line with findings from non-pregnant populations, which have shown that individuals with non-obesity and normal weight individuals who had metabolic profiles characteristic to obesity, and hence were metabolically unhealthy, were at increased risk of type 2 diabetes, all-cause mortality [19], and cardiovascular disease [41]. On the other hand, high BMI may indicate high lean body mass, which mostly consists of muscle. Fat and muscle have different metabolic characteristics, which may explain why some women with high BMI may have low values of BMI-related PMRSs.

In addition to being an indicator of health status, BMI also represents a social phenomenon associated with a number of social, economic and lifestyle factors [42]. These social, economic and lifestyle factors have an impact on mother–child outcomes examined in this study [43, 44]. However, our findings were not explained by maternal age, parity, level of education or substance use, which can be considered as crude proxies of maternal socio-economic status and lifestyle. This may suggest that the application of the supervised analytic method allowed us to disentangle metabolome-related aspect of BMI from an aspect of BMI that reflects social, economic and lifestyle factors.

Our study may bear clinical relevance. However, reference values of metabolic measures of normal weight, normotensive and normoglycemic women, which were used as a basis for generating the BMI-related PMRSs, need to be verified in larger samples of pregnant women, and corrected for gestational age. Moreover, future studies are needed to optimize the scaling of the metabolic measures, namely the extent to which the individual values are falling outside of the referent range. However, our approach of using metabolite values falling outside the upper and lower 5th percentiles allows replicating the findings in other cohorts and increases the clinical utility of the findings. This would not have been possible if we had standardized the metabolic measures, as the values would always be sample-specific. Scaling using raw values of the metabolic measures would not have been a possibility either, as the measurement scales of the metabolic measures vary, which would have translated into a larger contribution of measures with larger units into the PMRSs.

Our study has several strengths. First, as a predictor, we used the variation in the metabolome specific to the variation in maternal pre-pregnancy BMI and accounted for the correlated nature of metabolomics data. This allowed us to increase the specificity of the metabolome-defined BMI and understand and take into account the relative contributions of the individual metabolic measures in the metabolome-defined BMI. Second, we replicated most of the findings in an independent replication cohort, which differed in many characteristics from our initial study cohort, thus confirming the initial findings. Third, since the BMI-related PMRSs are based on reference values of metabolic measures in metabolically healthy pregnant mothers, and since the number of metabolic measures is limited to 57 in the PMRS with the lowest VIP threshold and to 14 in the PMRS with the highest VIP threshold, our findings are easy to replicate in other populations of pregnant mothers and their children. The metabolomic platform, which we used in this study, has been used in many epidemiological studies, and many of the metabolic measures included in the PMRSs have been clinically validated, which facilitates replication. As we have shown that all PMRSs were highly correlated and performed similarly in predicting mother–child adversities, the choice of 14 metabolic measures in resource-limited settings should be well justified. Other study strengths include the prospective study design, well-characterized samples, a large, targeted set of metabolites measured during pregnancy and from blood samples taken in the morning after a 10-h fast, and data on the mother–child outcomes extracted from nationwide registers and measured using validated tools. The limitation of the PREDO study is that the majority of the women were recruited based on their clinical risk factor status for preeclampsia resulting in overrepresentation of obesity and pregnancy complications in the sample. However, we tried to overcome this limitation by replicating the results in the independent ITU cohort where the prevalence of obesity and pregnancy complications was closer to the population prevalence. Children in the ITU cohort were younger than children in the PREDO cohort and at the time of follow-up, many of them were still too young to have been diagnosed with psychological developmental disorders, which may have affected the lack of replication of the findings with regard to child neurodevelopmental delay. Attrition was notable with regard to mother-reported child neurodevelopmental delay in both cohorts, which may have resulted in failure to detect children with milder delays in neurodevelopment than the diagnostic criteria require. A further limitation is that the study was conducted in a high-resource Nordic setting, which limits generalizability.

## Conclusions

Maternal BMI-defined metabolome and the novel, high maternal BMI-related PMRSs improve the prediction of maternal pregnancy complications, birth outcomes, and neurodevelopment in children over maternal BMI. The BMI-related PMRSs have the potential to become biomarkers identifying at-risk mothers and their children for timely targeted personalized interventions even at the level of maternal non-obesity and normal weight.

### Supplementary Information

**Additional file 1: Supplemental Table 1.** Inclusion and exclusion criteria for participation in the PREDO and ITU studies.

**Additional file 2: Supplemental Table 2.** Loadings of the metabolic measures on the maternal pre-pregnancy BMI-defined metabolome.

**Additional file 3: Supplemental Table 3.** Reference values of metabolic measures in normal weight, normoglycemic and normotensive women in PREDO.

**Additional file 4: Supplemental Table 4.** Associations of maternal BMI-defined metabolome and BMI-related PMRSs with maternal pregnancy complications, birth outcomes and child neurodevelopment in the subsample of women with normal weight.

**Additional file 5: Supplemental Figure 1.** Loadings of 95 metabolic measures on the maternal pre-pregnancy BMI-defined metabolome.

**Additional file 6: Supplemental Figure 2.** Loading of 95 metabolic measures on the maternal pre-pregnancy BMI at at each of the blood sampling points.

**Additional file 7: Supplemental Figure 3.** Metabolic measures included in each PMRS and their variable importance for the projection values.

**Additional file 8: Supplemental Figure 4.** Distributions of the PMRSs in women with normal weight, overweight and obesity.

**Additional file 9: Supplemental Material 1.** Step-by-step description of PMRS generation.

## Data Availability

PREDO and ITU data are confidential and protected by Finnish and EU laws. Collaboration in data analysis is possible through specific research proposals sent to the primary investigator Katri Räikkönen [katri.raikkonen@helsinki.fi].

## Abbreviations

AA: Amino acids
ASQ: Ages and Stages Questionnaires
Bayley III: Bayley Scales of Infant and Toddler Development, Third edition
BMI: Body Mass Index
CI: Confidence interval
FA: Fatty acids
GDM: Gestational diabetes mellitus
HILMO: Finnish Care Register for Health Care
ICD-10: International Statistical Classification of Diseases and Health-Related Problems 10th Revision
ITU: InTraUterine sampling in early pregnancy
IQR: Interquartile range
LDL: Low density lipoproteins
LGA: Large-for-gestational-age
MBR: Medical Birth Register
NICU: Neonatal intensive care unit
O-PLS: Orthogonal Partial Least Squares
OR: Odds ratio
PMRS: Polymetabolic risk score
RR: Relative risk
PREDO: Prediction and Prevention of Pre-eclampsia and Intrauterine Growth Restriction
SD: Standard deviation
SGA: Small-for-gestational-age
VIP: Variable importance for the projection
VLDL: Very low density lipoproteins
