# Supplementary material for: Associations of polymetabolic risk of high maternal pre-pregnancy body mass index with pregnancy complications, birth outcomes, and early childhood neurodevelopment: findings from two pregnancy cohorts
Source: BMC Pregnancy Childbirth. 2024 Jan 24;24:78. doi: 10.1186/s12884-024-06274-9 (PMC10807109; doi:10.1186/s12884-024-06274-9)
Supplement: Supplementary file 1 — Additional file 1: Supplemental Table 1. Inclusion and exclusion criteria for participation in the PREDO and ITU studies. [file 12884_2024_6274_MOESM1_ESM.docx]

Supplemental Table 1. Inclusion and exclusion criteria for participation in the PREDO and ITU studies

| PREDO | | ITU | |
| --- | --- | --- | --- |
| Inclusion criteria | Exclusion criteria | Inclusion criteria | Exclusion criteria |
| Sufficient Finnish  language ability to ensure informed consent | Multiple pregnancy | Sufficient Finnish  language ability to ensure informed consent | Multiple pregnancy |
| Singleton pregnancy | Asthma diagnosed by a physician | Singleton pregnancy | Maternal age younger than 18 years |
| First ultrasound screening at 12-13 of gestation | Allergy to acetylsalicylic acid | No diagnosis of chromosomal  abnormality | Fetal  chromosomal abnormalities |
| Known risk status for pre-eclampsia and IUGR:   1. Preeclampsia in previous pregnancy 2. Intrauterine growth restriction in previous pregnancy 3. Gestational diabetes in previous pregnancy 4. Pre-pregnancy obesity (body mass index  ≥ 30 kg/m2) 5. Chronic hypertension 6. Type 1 diabetes 7. Maternal age at childbirth below 20 years or above 40 years 8. Systemic lupus erythematosus 9. Sjögren’s syndrome 10. Previous pregnancy with fetal demise (> 22 gestational weeks or over 500 g) 11. No known risk factors | Tobacco smoking during pregnancy | Maternal age≥18 years |  |
| Live-born child | Previous peptic ulcer | Live-born child |  |
|  | Previous placental ablation |  |  |
|  | Inflammatory bowel diseases |  |  |
|  | Haemophilia or thrombophilia |  |  |
