# Supplementary material for: Associations of polymetabolic risk of high maternal pre-pregnancy body mass index with pregnancy complications, birth outcomes, and early childhood neurodevelopment: findings from two pregnancy cohorts
Source: BMC Pregnancy Childbirth. 2024 Jan 24;24:78. doi: 10.1186/s12884-024-06274-9 (PMC10807109; doi:10.1186/s12884-024-06274-9)
Supplement: Supplementary file 5 — Additional file 5: Supplemental Figure 1. Loadings of 95 metabolic measures on the maternal pre-pregnancy BMI-defined metabolome. [file 12884_2024_6274_MOESM5_ESM.pptx]

## Slide 1
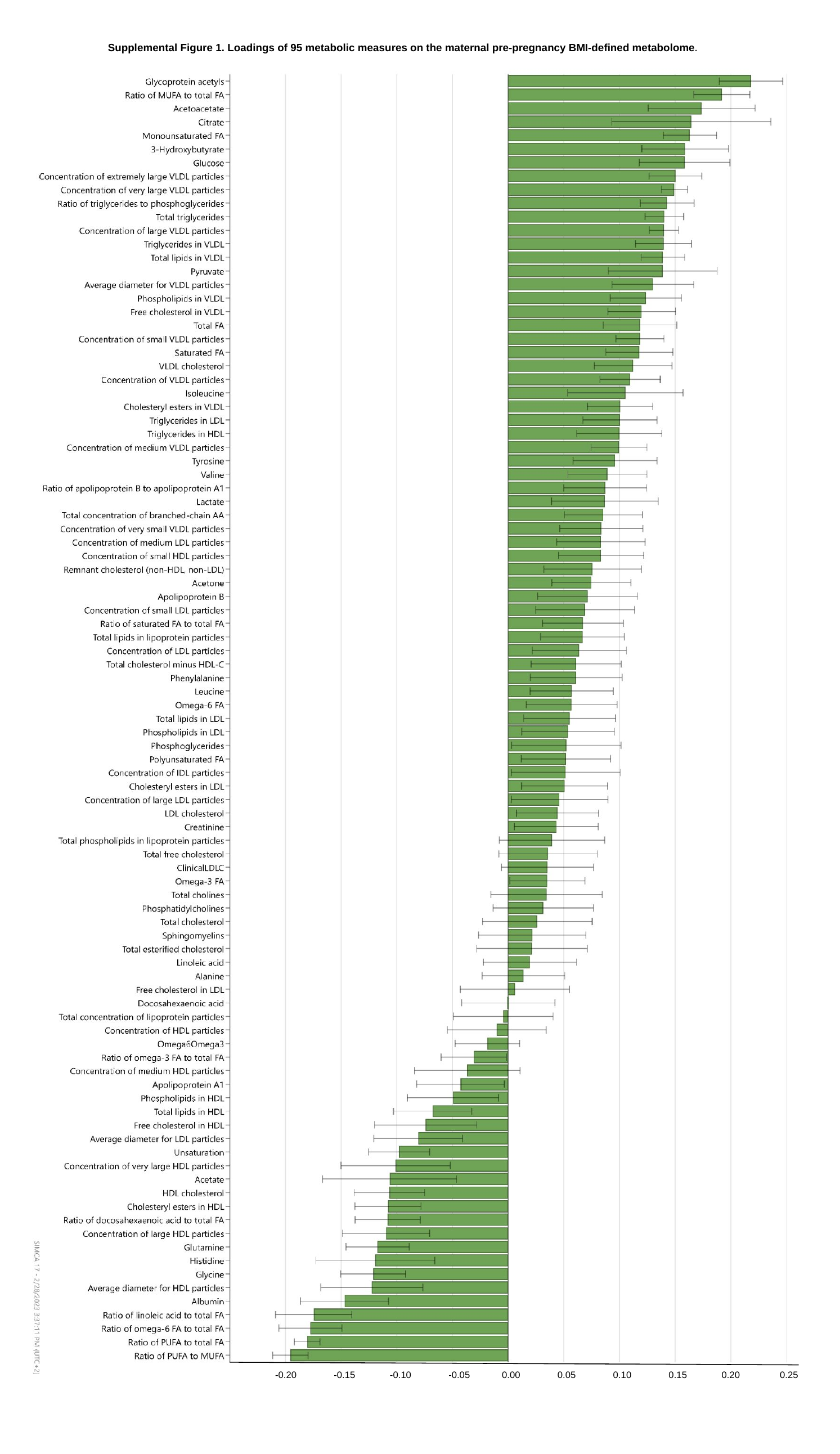

Supplemental Figure 1. Loadings of 95 metabolic measures on the maternal pre-pregnancy BMI-defined metabolome.
-0.20 -0.15 -0.10 -0.05 0.00 0.05 0.10 0.15 0.20 0.25
