# Supplementary material for: Associations of polymetabolic risk of high maternal pre-pregnancy body mass index with pregnancy complications, birth outcomes, and early childhood neurodevelopment: findings from two pregnancy cohorts
Source: BMC Pregnancy Childbirth. 2024 Jan 24;24:78. doi: 10.1186/s12884-024-06274-9 (PMC10807109; doi:10.1186/s12884-024-06274-9)
Supplement: Supplementary file 6 — Additional file 6: Supplemental Figure 2. Loading of 95 metabolic measures on the maternal pre-pregnancy BMI at at each of the blood sampling points. [file 12884_2024_6274_MOESM6_ESM.pptx]

## Slide 1
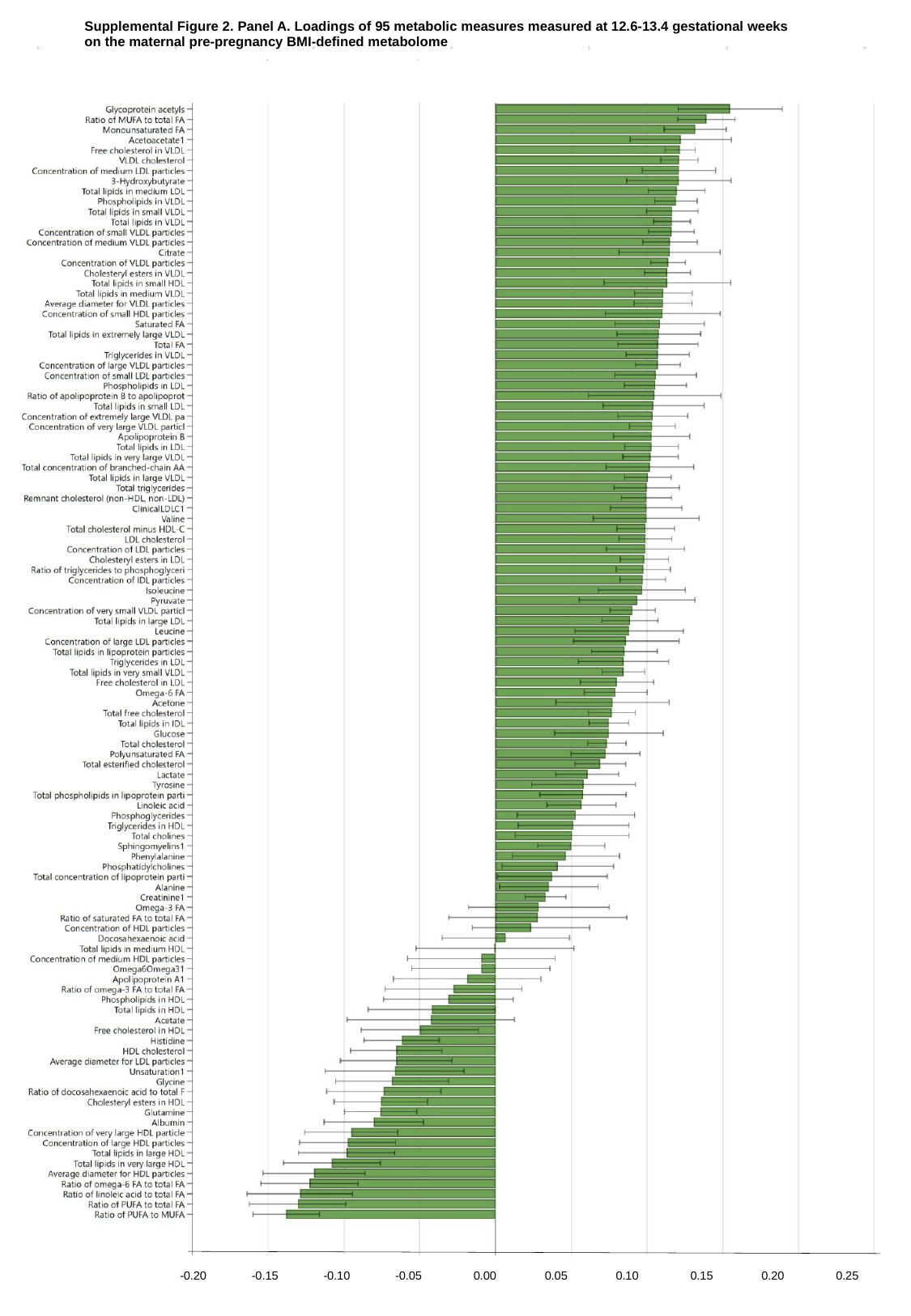

Supplemental Figure 2. Panel A. Loadings of 95 metabolic measures measured at 12.6-13.4 gestational weeks
on the maternal pre-pregnancy BMI-defined metabolome
-0.20 -0.15 -0.10 -0.05 0.00 0.05 0.10 0.15 0.20 0.25

## Slide 2
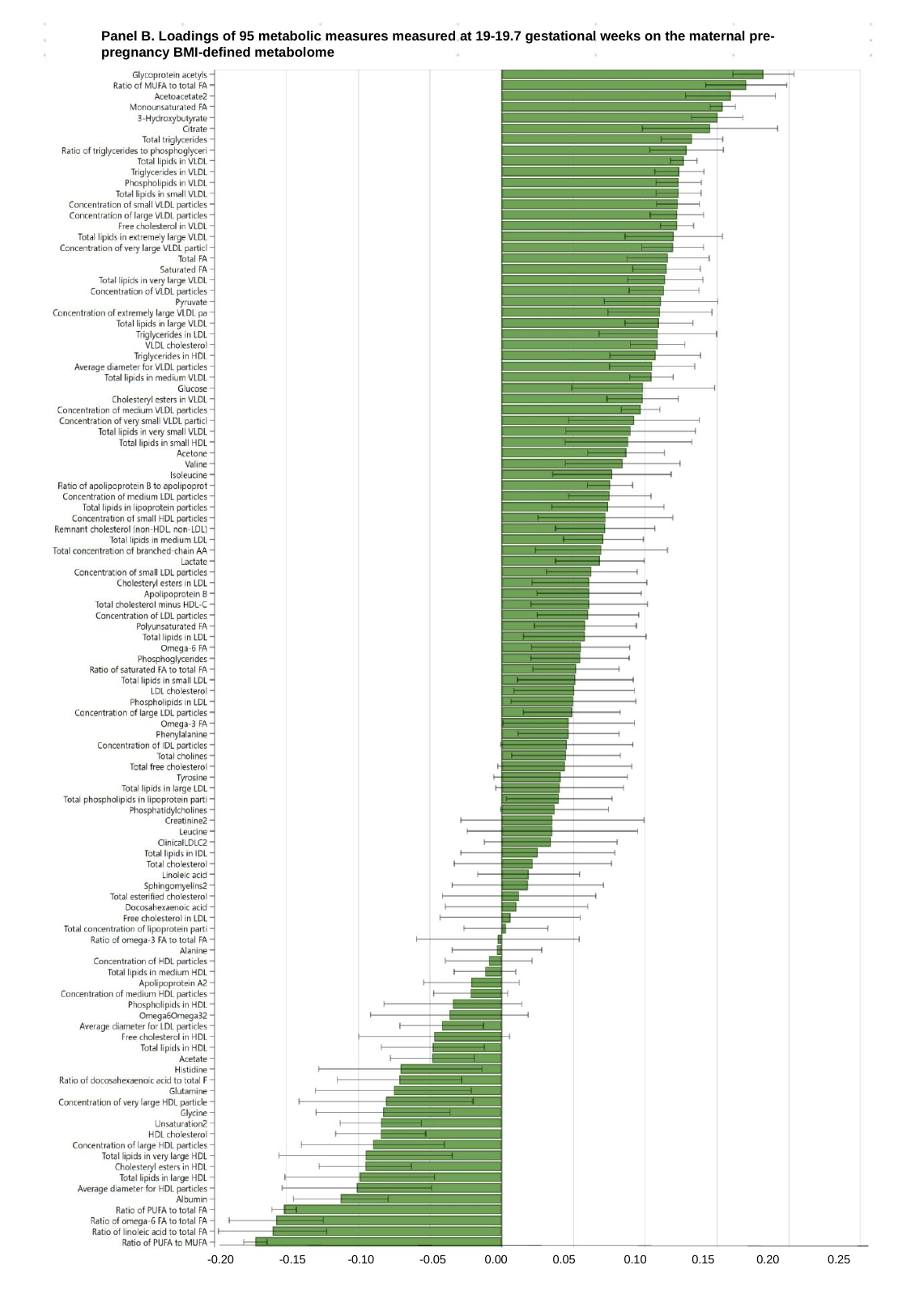

Panel B. Loadings of 95 metabolic measures measured at 19-19.7 gestational weeks on the maternal pre-pregnancy BMI-defined metabolome
-0.20 -0.15 -0.10 -0.05 0.00 0.05 0.10 0.15 0.20 0.25

## Slide 3
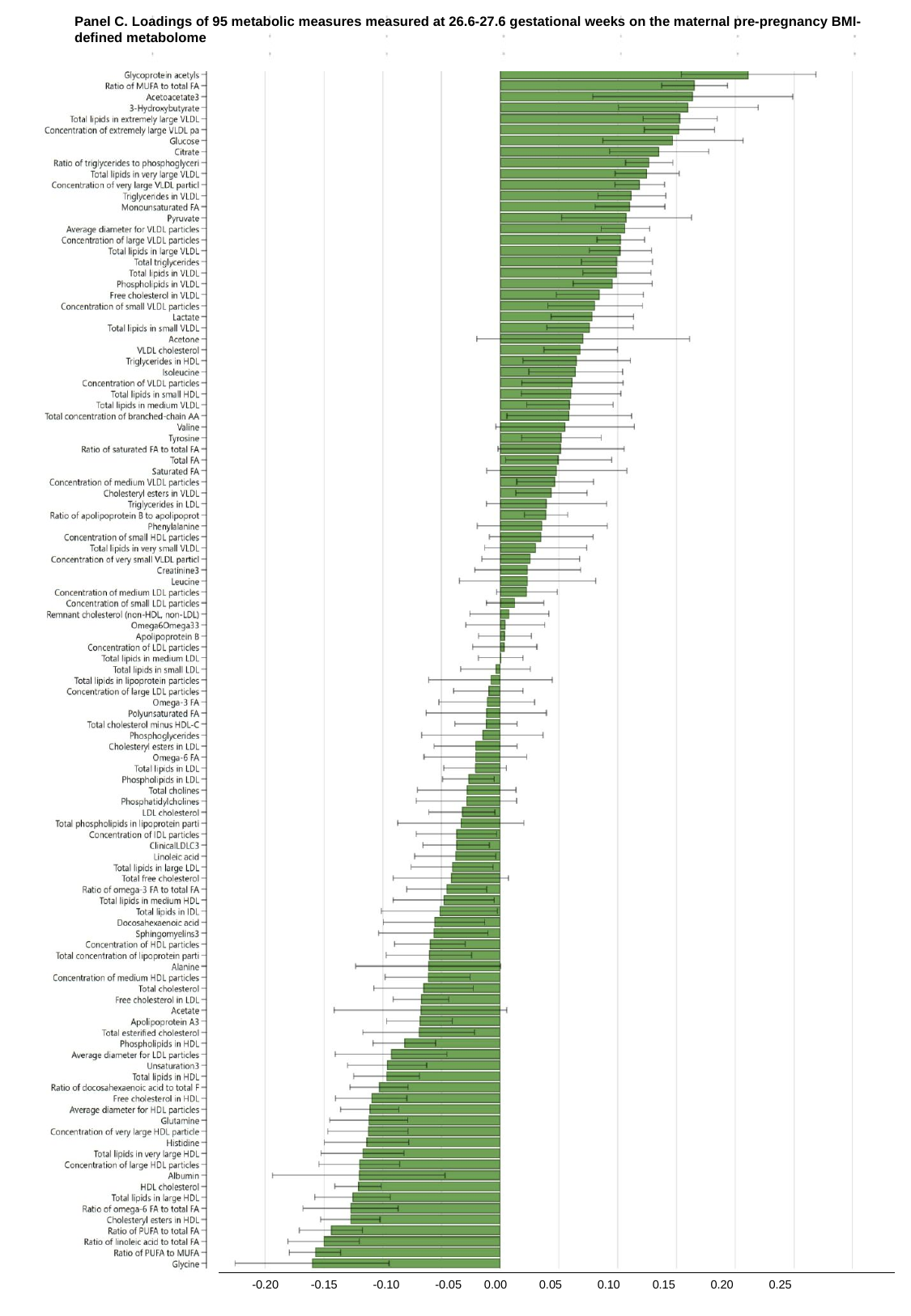

Panel C. Loadings of 95 metabolic measures measured at 26.6-27.6 gestational weeks on the maternal pre-pregnancy BMI-defined metabolome
-0.20 -0.15 -0.10 -0.05 0.00 0.05 0.10 0.15 0.20 0.25
