# Supplementary material for: Associations of polymetabolic risk of high maternal pre-pregnancy body mass index with pregnancy complications, birth outcomes, and early childhood neurodevelopment: findings from two pregnancy cohorts
Source: BMC Pregnancy Childbirth. 2024 Jan 24;24:78. doi: 10.1186/s12884-024-06274-9 (PMC10807109; doi:10.1186/s12884-024-06274-9)
Supplement: Supplementary file 7 — Additional file 7: Supplemental Figure 3. Metabolic measures included in each PMRS and their variable importance for the projection values. [file 12884_2024_6274_MOESM7_ESM.pptx]

## Slide 1
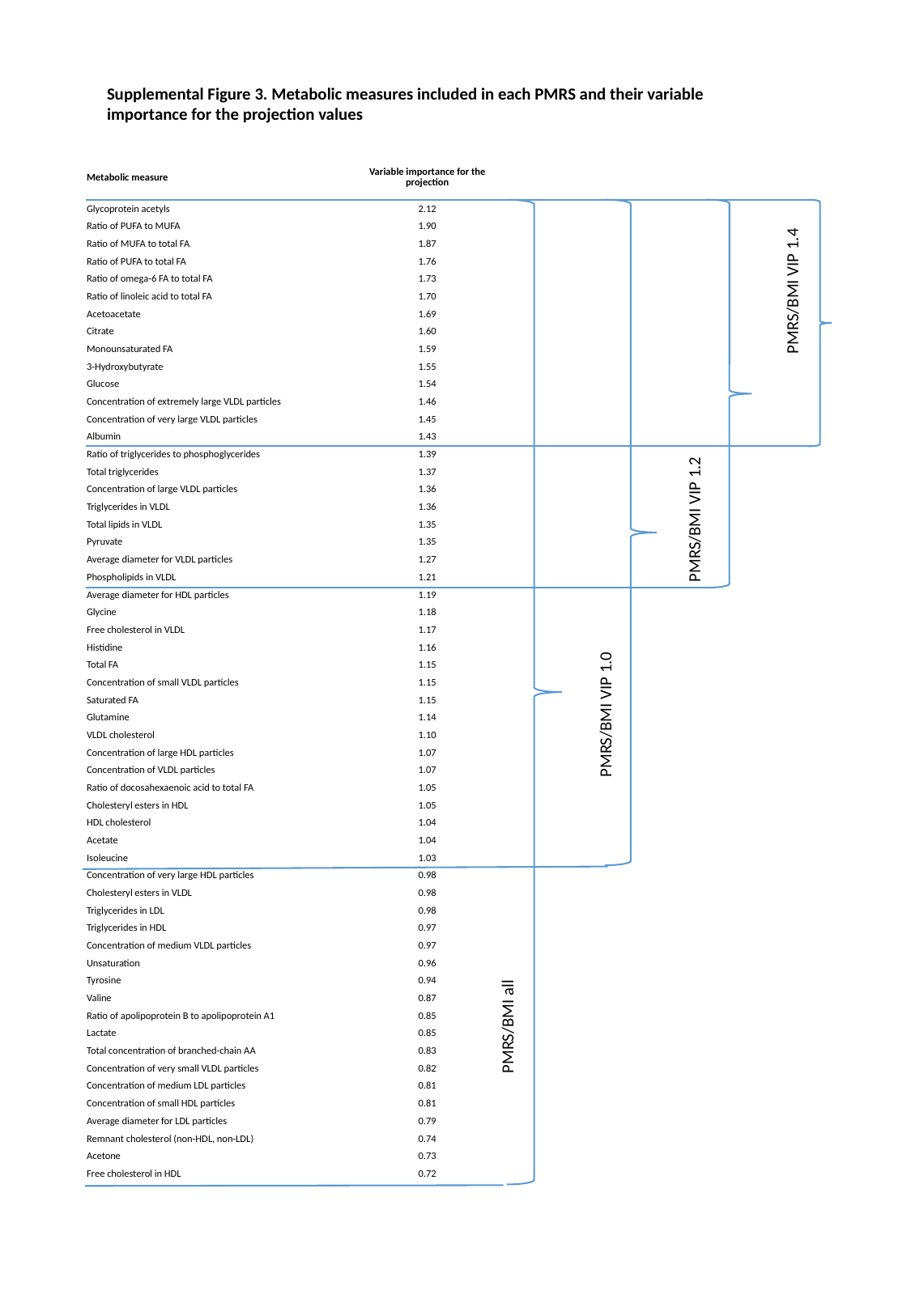

Supplemental Figure 3. Metabolic measures included in each PMRS and their variable
importance for the projection values
| Metabolic measure | Variable importance for the projection |
| --- | --- |
| Glycoprotein acetyls | 2.12 |
| Ratio of PUFA to MUFA | 1.90 |
| Ratio of MUFA to total FA | 1.87 |
| Ratio of PUFA to total FA | 1.76 |
| Ratio of omega-6 FA to total FA | 1.73 |
| Ratio of linoleic acid to total FA | 1.70 |
| Acetoacetate | 1.69 |
| Citrate | 1.60 |
| Monounsaturated FA | 1.59 |
| 3-Hydroxybutyrate | 1.55 |
| Glucose | 1.54 |
| Concentration of extremely large VLDL particles | 1.46 |
| Concentration of very large VLDL particles | 1.45 |
| Albumin | 1.43 |
| Ratio of triglycerides to phosphoglycerides | 1.39 |
| Total triglycerides | 1.37 |
| Concentration of large VLDL particles | 1.36 |
| Triglycerides in VLDL | 1.36 |
| Total lipids in VLDL | 1.35 |
| Pyruvate | 1.35 |
| Average diameter for VLDL particles | 1.27 |
| Phospholipids in VLDL | 1.21 |
| Average diameter for HDL particles | 1.19 |
| Glycine | 1.18 |
| Free cholesterol in VLDL | 1.17 |
| Histidine | 1.16 |
| Total FA | 1.15 |
| Concentration of small VLDL particles | 1.15 |
| Saturated FA | 1.15 |
| Glutamine | 1.14 |
| VLDL cholesterol | 1.10 |
| Concentration of large HDL particles | 1.07 |
| Concentration of VLDL particles | 1.07 |
| Ratio of docosahexaenoic acid to total FA | 1.05 |
| Cholesteryl esters in HDL | 1.05 |
| HDL cholesterol | 1.04 |
| Acetate | 1.04 |
| Isoleucine | 1.03 |
| Concentration of very large HDL particles | 0.98 |
| Cholesteryl esters in VLDL | 0.98 |
| Triglycerides in LDL | 0.98 |
| Triglycerides in HDL | 0.97 |
| Concentration of medium VLDL particles | 0.97 |
| Unsaturation | 0.96 |
| Tyrosine | 0.94 |
| Valine | 0.87 |
| Ratio of apolipoprotein B to apolipoprotein A1 | 0.85 |
| Lactate | 0.85 |
| Total concentration of branched-chain AA | 0.83 |
| Concentration of very small VLDL particles | 0.82 |
| Concentration of medium LDL particles | 0.81 |
| Concentration of small HDL particles | 0.81 |
| Average diameter for LDL particles | 0.79 |
| Remnant cholesterol (non-HDL, non-LDL) | 0.74 |
| Acetone | 0.73 |
| Free cholesterol in HDL | 0.72 |
PMRS/BMI VIP 1.4
PMRS/BMI VIP 1.2
PMRS/BMI VIP 1.0
PMRS/BMI all
