# Supplementary material for: Associations of polymetabolic risk of high maternal pre-pregnancy body mass index with pregnancy complications, birth outcomes, and early childhood neurodevelopment: findings from two pregnancy cohorts
Source: BMC Pregnancy Childbirth. 2024 Jan 24;24:78. doi: 10.1186/s12884-024-06274-9 (PMC10807109; doi:10.1186/s12884-024-06274-9)
Supplement: Supplementary file 8 — Additional file 8: Supplemental Figure 4. Distributions of the PMRSs in women with normal weight, overweight and obesity. [file 12884_2024_6274_MOESM8_ESM.pptx]

## Slide 1
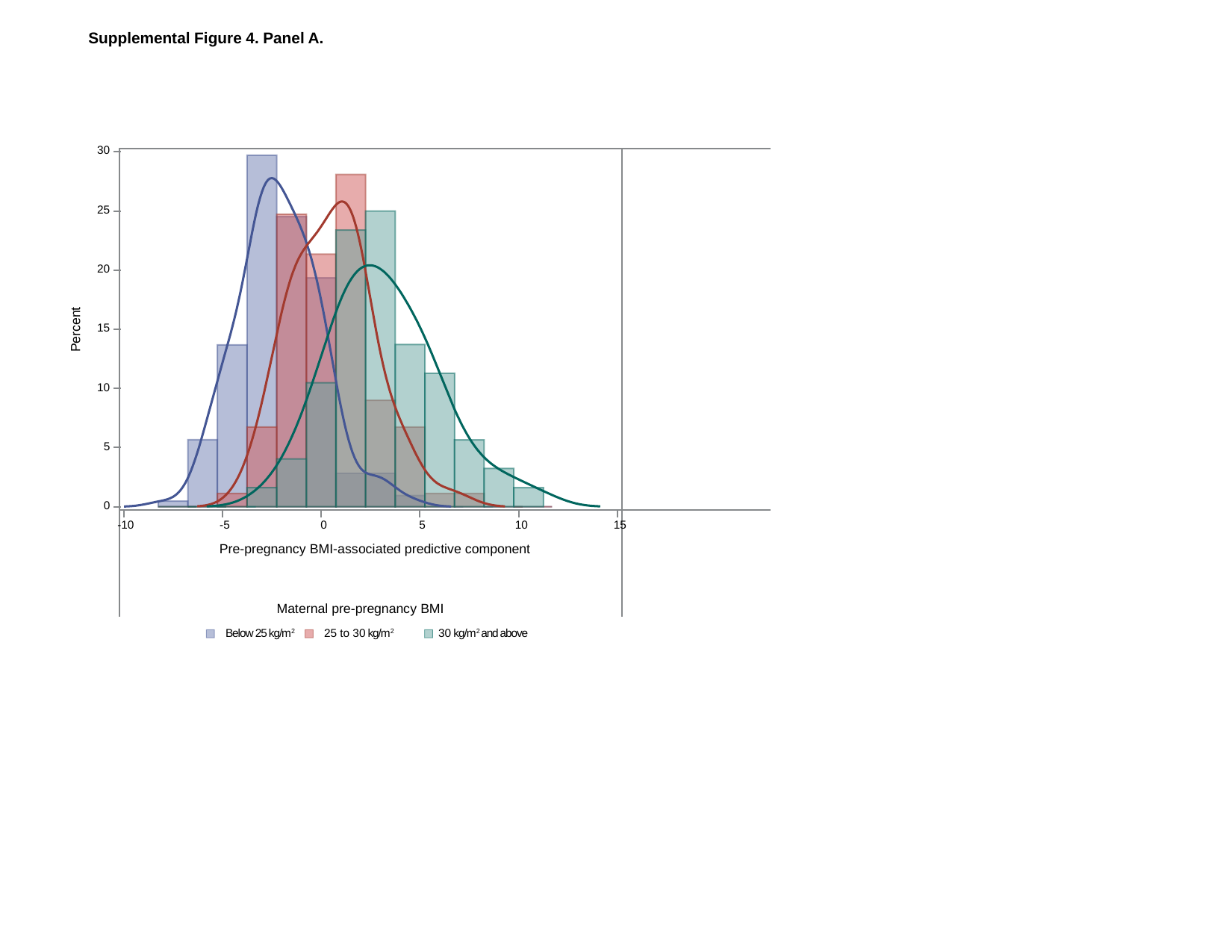

Supplemental Figure 4. Panel A.
30
25
20
15
Percent
10
5
0
-10	-5	0	5	10	15
Pre-pregnancy BMI-associated predictive component
Maternal pre-pregnancy BMI
Below 25 kg/m2	25 to 30 kg/m2 30 kg/m2 and above

## Slide 2
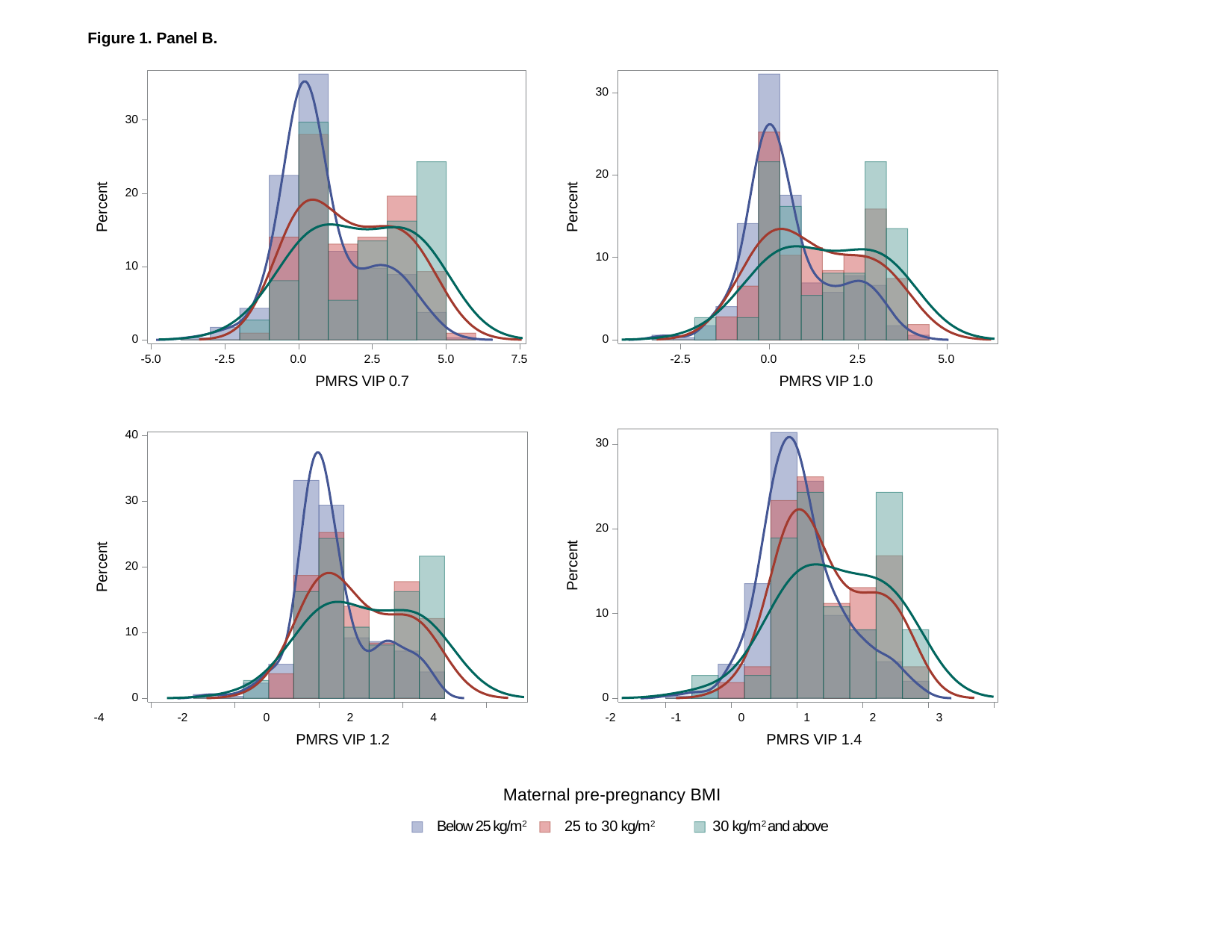

Figure 1. Panel B.
30
30
20
20
Percent
Percent
10
10
0
0
-5.0	-2.5	0.0	2.5	5.0	7.5
-2.5	0.0	2.5	5.0
PMRS VIP 0.7
PMRS VIP 1.0
40
30
30
20
Percent
Percent
20
10
10
0
0
-4	-2	0	2	4
-2	-1	0	1	2	3
PMRS VIP 1.2
PMRS VIP 1.4
Maternal pre-pregnancy BMI
Below 25 kg/m2	25 to 30 kg/m2 30 kg/m2 and above
