# Supplementary material for: Associations of polymetabolic risk of high maternal pre-pregnancy body mass index with pregnancy complications, birth outcomes, and early childhood neurodevelopment: findings from two pregnancy cohorts
Source: BMC Pregnancy Childbirth. 2024 Jan 24;24:78. doi: 10.1186/s12884-024-06274-9 (PMC10807109; doi:10.1186/s12884-024-06274-9)
Supplement: Supplementary file 9 — Additional file 9: Supplemental Material 1. Step-by-step description of PMRS generation. [file 12884_2024_6274_MOESM9_ESM.docx]

**Supplemental Material 1. Step-by-step description of PMRS generation**

From the predictive component extracted from O-PLS regression in the PREDO, we identified metabolic measures explaining variation in maternal early-pregnancy BMI and determined their importance for the projection (VIP). We created PMRS based on four sets of metabolites significantly contributing to the explanation of maternal BMI: all metabolic measures significantly contributing to the explanation of maternal BMI (PMRS all), metabolic measures with VIP 1.0 and higher (PMRS VIP 1.0), metabolic measures with VIP 1.2 and higher (PMRS VIP 1.2) and metabolic measures with VIP 1.4 and higher (PMRS VIP 1.4). The lists of metabolic measures included into each of the PMRS scores and corresponding VIP values are shown in the Supplemental Figure 3. To create PMRS replicable in the other samples, we first identified the range of metabolic measures’ values in normal weight women (18 kg/m2<BMI<25 kg/m^2^) without diabetic and hypertensive disorders in the PREDO cohort (n=131). Lower and higher boundaries of the range of reference values represented 5^th^ and 95^th^ percentile of metabolic measures’ values in normal weight women without diabetic and hypertensive disorders (Supplemental Table 3). Second, based on these reference values, we coded metabolic measures in a replication cohort as 0 if they were falling within, as -1 if they were below the lowest boundary, and as 1 if they were above the highest boundary of the reference range. We then used loadings of metabolic measures on predictive component from O-PLS model regressing maternal early-pregnancy BMI on 95 maternal metabolic measures during pregnancy in the PREDO as weights (Supplemental Table 2) and calculated PMRS scores by summing up the weights of 4 sets of metabolites described above (PMRS all, BMI/MRS VIP 1.0, PMRS VIP 1.2, PMRS VIP 1.4). We standardized the PMRS scores to the mean of 0 and SD of 1.

SAS code

**data** itu; set itu;

if TotalC <= **3.9472877** then TotalC =-**1**; else if **3.9472877** < TotalC < **6.6127125** then TotalC =**0**; else if TotalC >= **6.6127125** then TotalC =**1**;

if nonHDLC <= **2.2724413** then nonHDLC =-**1**; else if **2.2724413** < nonHDLC < **4.7124545** then nonHDLC =**0**; else if nonHDLC >= **4.7124545** then nonHDLC =**1**;

if RemnantC <= **1.0782613** then RemnantC =-**1**; else if **1.0782613** < RemnantC < **2.3023423** then RemnantC =**0**; else if RemnantC >= **2.3023423** then RemnantC =**1**;

if VLDLC <= **0.3978686** then VLDLC =-**1**; else if **0.3978686** < VLDLC < **0.9815272** then VLDLC =**0**; else if VLDLC >= **0.9815272** then VLDLC =**1**;

if ClinicalLDLC <= **1.5780591** then ClinicalLDLC =-**1**; else if **1.5780591** < ClinicalLDLC < **3.6139491** then ClinicalLDLC =**0**; else if ClinicalLDLC >= **3.6139491** then ClinicalLDLC =**1**;

if LDLC <= **1.2017413** then LDLC =-**1**; else if **1.2017413** < LDLC < **2.4082689** then LDLC =**0**; else if LDLC >= **2.4082689** then LDLC =**1**;

if HDLC <= **1.3681271** then HDLC =-**1**; else if **1.3681271** < HDLC < **2.2767110** then HDLC =**0**; else if HDLC >= **2.2767110** then HDLC =**1**;

if TotalTG <= **0.8069831** then TotalTG =-**1**; else if **0.8069831** < TotalTG < **1.9827235** then TotalTG =**0**; else if TotalTG >= **1.9827235** then TotalTG =**1**;

if VLDLTG <= **0.3897376** then VLDLTG =-**1**; else if **0.3897376** < VLDLTG < **1.2196320** then VLDLTG =**0**; else if VLDLTG >= **1.2196320** then VLDLTG =**1**;

if LDLTG <= **0.1413444** then LDLTG =-**1**; else if **0.1413444** < LDLTG < **0.2831056** then LDLTG =**0**; else if LDLTG >= **0.2831056** then LDLTG =**1**;

if HDLTG <= **0.1451646** then HDLTG =-**1**; else if **0.1451646** < HDLTG < **0.3096777** then HDLTG =**0**; else if HDLTG >= **0.3096777** then HDLTG =**1**;

if TotalPL <= **2.7243756** then TotalPL =-**1**; else if **2.7243756** < TotalPL < **4.2081449** then TotalPL =**0**; else if TotalPL >= **4.2081449** then TotalPL =**1**;

if VLDLPL <= **0.2456294** then VLDLPL =-**1**; else if **0.2456294** < VLDLPL < **0.6320528** then VLDLPL =**0**; else if VLDLPL >= **0.6320528** then VLDLPL =**1**;

if LDLPL <= **0.4177797** then LDLPL =-**1**; else if **0.4177797** < LDLPL < **0.8073030** then LDLPL =**0**; else if LDLPL >= **0.8073030** then LDLPL =**1**;

if HDLPL <= **1.6435264** then HDLPL =-**1**; else if **1.6435264** < HDLPL < **2.5786967** then HDLPL =**0**; else if HDLPL >= **2.5786967** then HDLPL =**1**;

if TotalCE <= **2.8701645** then TotalCE =-**1**; else if **2.8701645** < TotalCE < **4.7678274** then TotalCE =**0**; else if TotalCE >= **4.7678274** then TotalCE =**1**;

if VLDLCE <= **0.2386583** then VLDLCE =-**1**; else if **0.2386583** < VLDLCE < **0.5858779** then VLDLCE =**0**; else if VLDLCE >= **0.5858779** then VLDLCE =**1**;

if LDLCE <= **0.8670357** then LDLCE =-**1**; else if **0.8670357** < LDLCE < **1.7507470** then LDLCE =**0**; else if LDLCE >= **1.7507470** then LDLCE =**1**;

if HDLCE <= **1.0331995** then HDLCE =-**1**; else if **1.0331995** < HDLCE < **1.6836391** then HDLCE =**0**; else if HDLCE >= **1.6836391** then HDLCE =**1**;

if TotalFC <= **1.0281725** then TotalFC =-**1**; else if **1.0281725** < TotalFC < **1.8264581** then TotalFC =**0**; else if TotalFC >= **1.8264581** then TotalFC =**1**;

if VLDLFC <= **0.1484137** then VLDLFC =-**1**; else if **0.1484137** < VLDLFC < **0.3883934** then VLDLFC =**0**; else if VLDLFC >= **0.3883934** then VLDLFC =**1**;

if LDLFC <= **0.3208060** then LDLFC =-**1**; else if **0.3208060** < LDLFC < **0.6537348** then LDLFC =**0**; else if LDLFC >= **0.6537348** then LDLFC =**1**;

if HDLFC <= **0.3436197** then HDLFC =-**1**; else if **0.3436197** < HDLFC < **0.5710359** then HDLFC =**0**; else if HDLFC >= **0.5710359** then HDLFC =**1**;

if TotalL <= **7.6166938** then TotalL =-**1**; else if **7.6166938** < TotalL < **12.3314403** then TotalL =**0**; else if TotalL >= **12.3314403** then TotalL =**1**;

if VLDLL <= **1.0166578** then VLDLL =-**1**; else if **1.0166578** < VLDLL < **2.7476890** then VLDLL =**0**; else if VLDLL >= **2.7476890** then VLDLL =**1**;

if LDLL <= **1.7553213** then LDLL =-**1**; else if **1.7553213** < LDLL < **3.5000359** then LDLL =**0**; else if LDLL >= **3.5000359** then LDLL =**1**;

if HDLL <= **3.1972734** then HDLL =-**1**; else if **3.1972734** < HDLL < **5.1552032** then HDLL =**0**; else if HDLL >= **5.1552032** then HDLL =**1**;

if TotalP <= **0.0157969** then TotalP =-**1**; else if **0.0157969** < TotalP < **0.0227961** then TotalP =**0**; else if TotalP >= **0.0227961** then TotalP =**1**;

if VLDLP <= **0.000101139** then VLDLP =-**1**; else if **0.000101139** < VLDLP < **0.000214013** then VLDLP =**0**; else if VLDLP >= **0.000214013** then VLDLP =**1**;

if LDLP <= **0.000789318** then LDLP =-**1**; else if **0.000789318** < LDLP < **0.0015682** then LDLP =**0**; else if LDLP >= **0.0015682** then LDLP =**1**;

if HDLP <= **0.0143765** then HDLP =-**1**; else if **0.0143765** < HDLP < **0.0211305** then HDLP =**0**; else if HDLP >= **0.0211305** then HDLP =**1**;

if VLDLsize <= **35.9645630** then VLDLsize =-**1**; else if **35.9645630** < VLDLsize < **38.5795570** then VLDLsize =**0**; else if VLDLsize >= **38.5795570** then VLDLsize =**1**;

if LDLsize <= **23.8505715** then LDLsize =-**1**; else if **23.8505715** < LDLsize < **24.0299633** then LDLsize =**0**; else if LDLsize >= **24.0299633** then LDLsize =**1**;

if HDLsize <= **9.7625870** then HDLsize =-**1**; else if **9.7625870** < HDLsize < **10.3778860** then HDLsize =**0**; else if HDLsize >= **10.3778860** then HDLsize =**1**;

if Phosphoglyc <= **2.2564738** then Phosphoglyc =-**1**; else if **2.2564738** < Phosphoglyc < **3.4679766** then Phosphoglyc =**0**; else if Phosphoglyc >= **3.4679766** then Phosphoglyc =**1**;

if TGPG <= **0.3241690** then TGPG =-**1**; else if **0.3241690** < TGPG < **0.6013649** then TGPG =**0**; else if TGPG >= **0.6013649** then TGPG =**1**;

if Cholines <= **2.5223627** then Cholines =-**1**; else if **2.5223627** < Cholines < **3.8033475** then Cholines =**0**; else if Cholines >= **3.8033475** then Cholines =**1**;

if Phosphatidylc <= **2.1936047** then Phosphatidylc =-**1**; else if **2.1936047** < Phosphatidylc < **3.3708190** then Phosphatidylc =**0**; else if Phosphatidylc >= **3.3708190** then Phosphatidylc =**1**;

if Sphingomyelins <= **0.4270021** then Sphingomyelins =-**1**; else if **0.4270021** < Sphingomyelins < **0.6563493** then Sphingomyelins =**0**; else if Sphingomyelins >= **0.6563493** then Sphingomyelins =**1**;

if ApoB <= **0.5831033** then ApoB =-**1**; else if **0.5831033** < ApoB < **1.1375696** then ApoB =**0**; else if ApoB >= **1.1375696** then ApoB =**1**;

if ApoA1 <= **1.4746470** then ApoA1 =-**1**; else if **1.4746470** < ApoA1 < **2.2247854** then ApoA1 =**0**; else if ApoA1 >= **2.2247854** then ApoA1 =**1**;

if ApoBApoA1 <= **0.3087561** then ApoBApoA1 =-**1**; else if **0.3087561** < ApoBApoA1 < **0.6495905** then ApoBApoA1 =**0**; else if ApoBApoA1 >= **0.6495905** then ApoBApoA1 =**1**;

if TotalFA <= **10.0703873** then TotalFA =-**1**; else if **10.0703873** < TotalFA < **16.4206580** then TotalFA =**0**; else if TotalFA >= **16.4206580** then TotalFA =**1**;

if Unsaturation <= **1.2746987** then Unsaturation =-**1**; else if **1.2746987** < Unsaturation < **1.4412538** then Unsaturation =**0**; else if Unsaturation >= **1.4412538** then Unsaturation =**1**;

if Omega3 <= **0.3323908** then Omega3 =-**1**; else if **0.3323908** < Omega3 < **1.0065131** then Omega3 =**0**; else if Omega3 >= **1.0065131** then Omega3 =**1**;

if Omega6 <= **3.9198366** then Omega6 =-**1**; else if **3.9198366** < Omega6 < **5.9142708** then Omega6 =**0**; else if Omega6 >= **5.9142708** then Omega6 =**1**;

if PUFA <= **4.3971898** then PUFA =-**1**; else if **4.3971898** < PUFA < **6.8288750** then PUFA =**0**; else if PUFA >= **6.8288750** then PUFA =**1**;

if MUFA <= **2.1701967** then MUFA =-**1**; else if **2.1701967** < MUFA < **4.0863059** then MUFA =**0**; else if MUFA >= **4.0863059** then MUFA =**1**;

if SFA <= **3.5237054** then SFA =-**1**; else if **3.5237054** < SFA < **6.1635968** then SFA =**0**; else if SFA >= **6.1635968** then SFA =**1**;

if LA <= **2.9531908** then LA =-**1**; else if **2.9531908** < LA < **4.9489112** then LA =**0**; else if LA >= **4.9489112** then LA =**1**;

if DHA <= **0.2029141** then DHA =-**1**; else if **0.2029141** < DHA < **0.4431653** then DHA =**0**; else if DHA >= **0.4431653** then DHA =**1**;

if Omega3_A <= **2.7964333** then Omega3_A =-**1**; else if **2.7964333** < Omega3_A < **6.8764815** then Omega3_A =**0**; else if Omega3_A >= **6.8764815** then Omega3_A =**1**;

if Omega6_A <= **33.8285833** then Omega6_A =-**1**; else if **33.8285833** < Omega6_A < **39.9453795** then Omega6_A =**0**; else if Omega6_A >= **39.9453795** then Omega6_A =**1**;

if PUFA_A <= **38.3760650** then PUFA_A =-**1**; else if **38.3760650** < PUFA_A < **44.8207180** then PUFA_A =**0**; else if PUFA_A >= **44.8207180** then PUFA_A =**1**;

if MUFA_A <= **20.1673000** then MUFA_A =-**1**; else if **20.1673000** < MUFA_A < **24.8696943** then MUFA_A =**0**; else if MUFA_A >= **24.8696943** then MUFA_A =**1**;

if SFA_A <= **33.7547023** then SFA_A =-**1**; else if **33.7547023** < SFA_A < **37.7984627** then SFA_A =**0**; else if SFA_A >= **37.7984627** then SFA_A =**1**;

if LA_A <= **26.4281160** then LA_A =-**1**; else if **26.4281160** < LA_A < **32.8011520** then LA_A =**0**; else if LA_A >= **32.8011520** then LA_A =**1**;

if DHA_A <= **1.6778340** then DHA_A =-**1**; else if **1.6778340** < DHA_A < **3.2034684** then DHA_A =**0**; else if DHA_A >= **3.2034684** then DHA_A =**1**;

if PUFAMUFA <= **1.5699859** then PUFAMUFA =-**1**; else if **1.5699859** < PUFAMUFA < **2.2548982** then PUFAMUFA =**0**; else if PUFAMUFA >= **2.2548982** then PUFAMUFA =**1**;

if Omega6Omega3 <= **5.3197107** then Omega6Omega3 =-**1**; else if **5.3197107** < Omega6Omega3 < **14.0142413** then Omega6Omega3 =**0**; else if Omega6Omega3 >= **14.0142413** then Omega6Omega3 =**1**;

if Ala <= **0.2213823** then Ala =-**1**; else if **0.2213823** < Ala < **0.3615827** then Ala =**0**; else if Ala >= **0.3615827** then Ala =**1**;

if Gln <= **0.3754476** then Gln =-**1**; else if **0.3754476** < Gln < **0.5121330** then Gln =**0**; else if Gln >= **0.5121330** then Gln =**1**;

if Gly <= **0.0666475** then Gly =-**1**; else if **0.0666475** < Gly < **0.1699205** then Gly =**0**; else if Gly >= **0.1699205** then Gly =**1**;

if His <= **0.0584374** then His =-**1**; else if **0.0584374** < His < **0.0797270** then His =**0**; else if His >= **0.0797270** then His =**1**;

if TotalBCAA <= **0.2345398** then TotalBCAA =-**1**; else if **0.2345398** < TotalBCAA < **0.3242063** then TotalBCAA =**0**; else if TotalBCAA >= **0.3242063** then TotalBCAA =**1**;

if Ile <= **0.0291795** then Ile =-**1**; else if **0.0291795** < Ile < **0.0447497** then Ile =**0**; else if Ile >= **0.0447497** then Ile =**1**;

if Leu <= **0.0553023** then Leu =-**1**; else if **0.0553023** < Leu < **0.0832339** then Leu =**0**; else if Leu >= **0.0832339** then Leu =**1**;

if Val <= **0.1468304** then Val =-**1**; else if **0.1468304** < Val < **0.2009373** then Val =**0**; else if Val >= **0.2009373** then Val =**1**;

if Phe <= **0.0362943** then Phe =-**1**; else if **0.0362943** < Phe < **0.0480252** then Phe =**0**; else if Phe >= **0.0480252** then Phe =**1**;

if Tyr <= **0.0374816** then Tyr =-**1**; else if **0.0374816** < Tyr < **0.0556760** then Tyr =**0**; else if Tyr >= **0.0556760** then Tyr =**1**;

if Glucose <= **4.3067507** then Glucose =-**1**; else if **4.3067507** < Glucose < **5.2571507** then Glucose =**0**; else if Glucose >= **5.2571507** then Glucose =**1**;

if Lactate <= **0.6603919** then Lactate =-**1**; else if **0.6603919** < Lactate < **1.4930800** then Lactate =**0**; else if Lactate >= **1.4930800** then Lactate =**1**;

if Pyruvate <= **0.0208237** then Pyruvate =-**1**; else if **0.0208237** < Pyruvate < **0.0841120** then Pyruvate =**0**; else if Pyruvate >= **0.0841120** then Pyruvate =**1**;

if Citrate <= **0.0456841** then Citrate =-**1**; else if **0.0456841** < Citrate < **0.0683380** then Citrate =**0**; else if Citrate >= **0.0683380** then Citrate =**1**;

if bOHbutyrate <= **0.0171106** then bOHbutyrate =-**1**; else if **0.0171106** < bOHbutyrate < **0.1152035** then bOHbutyrate =**0**; else if bOHbutyrate >= **0.1152035** then bOHbutyrate =**1**;

if Acetate <= **0.0276081** then Acetate =-**1**; else if **0.0276081** < Acetate < **0.0853966** then Acetate =**0**; else if Acetate >= **0.0853966** then Acetate =**1**;

if Acetoacetate <= **0.0100460** then Acetoacetate =-**1**; else if **0.0100460** < Acetoacetate < **0.0471720** then Acetoacetate =**0**; else if Acetoacetate >= **0.0471720** then Acetoacetate =**1**;

if Acetone <= **0.0111248** then Acetone =-**1**; else if **0.0111248** < Acetone < **0.0201130** then Acetone =**0**; else if Acetone >= **0.0201130** then Acetone =**1**;

if Creatinine <= **0.0450114** then Creatinine =-**1**; else if **0.0450114** < Creatinine < **0.0640351** then Creatinine =**0**; else if Creatinine >= **0.0640351** then Creatinine =**1**;

if Albumin <= **29.3660740** then Albumin =-**1**; else if **29.3660740** < Albumin < **35.1757390** then Albumin =**0**; else if Albumin >= **35.1757390** then Albumin =**1**;

if GlycA <= **0.7142434** then GlycA =-**1**; else if **0.7142434** < GlycA < **0.9381353** then GlycA =**0**; else if GlycA >= **0.9381353** then GlycA =**1**;

if IDLP <= **0.000234754** then IDLP =-**1**; else if **0.000234754** < IDLP < **0.000462787** then IDLP =**0**; else if IDLP >= **0.000462787** then IDLP =**1**;

if LHDLP <= **0.0017211** then LHDLP =-**1**; else if **0.0017211** < LHDLP < **0.0044452** then LHDLP =**0**; else if LHDLP >= **0.0044452** then LHDLP =**1**;

if LLDLP <= **0.000506222** then LLDLP =-**1**; else if **0.000506222** < LLDLP < **0.000969346** then LLDLP =**0**; else if LLDLP >= **0.000969346** then LLDLP =**1**;

if LVLDLP <= **3.309402E-6** then LVLDLP =-**1**; else if **3.309402E-6** < LVLDLP < **0.000013687** then LVLDLP =**0**; else if LVLDLP >= **0.000013687** then LVLDLP =**1**;

if MHDLP <= **0.0039106** then MHDLP =-**1**; else if **0.0039106** < MHDLP < **0.0064379** then MHDLP =**0**; else if MHDLP >= **0.0064379** then MHDLP =**1**;

if MLDLP <= **0.000174608** then MLDLP =-**1**; else if **0.000174608** < MLDLP < **0.000382131** then MLDLP =**0**; else if MLDLP >= **0.000382131** then MLDLP =**1**;

if MVLDLP <= **0.000017145** then MVLDLP =-**1**; else if **0.000017145** < MVLDLP < **0.000046167** then MVLDLP =**0**; else if MVLDLP >= **0.000046167** then MVLDLP =**1**;

if SHDLP <= **0.0073732** then SHDLP =-**1**; else if **0.0073732** < SHDLP < **0.0110510** then SHDLP =**0**; else if SHDLP >= **0.0110510** then SHDLP =**1**;

if SLDLP <= **0.000118072** then SLDLP =-**1**; else if **0.000118072** < SLDLP < **0.000214469** then SLDLP =**0**; else if SLDLP >= **0.000214469** then SLDLP =**1**;

if SVLDLP <= **0.000026363** then SVLDLP =-**1**; else if **0.000026363** < SVLDLP < **0.000059590** then SVLDLP =**0**; else if SVLDLP >= **0.000059590** then SVLDLP =**1**;

if XLHDLP <= **0.000279138** then XLHDLP =-**1**; else if **0.000279138** < XLHDLP < **0.000684871** then XLHDLP =**0**; else if XLHDLP >= **0.000684871** then XLHDLP =**1**;

if XLVLDLP <= **0.00000063844372** then XLVLDLP =-**1**; else if **0.00000063844372** < XLVLDLP < **0.0000044422855** then XLVLDLP =**0**; else if XLVLDLP >= **0.0000044422855** then XLVLDLP =**1**;

if XSVLDLP <= **0.000050108** then XSVLDLP =-**1**; else if **0.000050108** < XSVLDLP < **0.000094178** then XSVLDLP =**0**; else if XSVLDLP >= **0.000094178** then XSVLDLP =**1**;

if XXLVLDLP <= **0.00000012972883** then XXLVLDLP =-**1**; else if **0.00000012972883** < XXLVLDLP < **0.0000020563448** then XXLVLDLP =**0**; else if XXLVLDLP >= **0.0000020563448** then XXLVLDLP =**1**;

**run**;

**data** itu; set itu;

mrs_vip1=

( GlycA * **0.217996** )+

( PUFAMUFA * -**0.195448** )+

( MUFA_A * **0.192041** )+

( PUFA_A * -**0.180506** )+

( Omega6_A * -**0.177534** )+

( LA_A * -**0.174586** )+

( Acetoacetate * **0.173698** )+

( Citrate * **0.164528** )+

( MUFA * **0.163087** )+

( bOHbutyrate * **0.158902** )+

( Glucose * **0.15851** )+

( XXLVLDLP * **0.150205** )+

( XLVLDLP * **0.149226** )+

( Albumin * -**0.146904** )+

( TGPG * **0.142717** )+

( TotalTG * **0.140311** )+

( LVLDLP * **0.139834** )+

( VLDLTG * **0.139651** )+

( VLDLL * **0.138936** )+

( Pyruvate * **0.138877** )+

( VLDLsize * **0.130065** )+

( VLDLPL * **0.123675** )+

( HDLsize * -**0.122485** )+

( Gly * -**0.121222** )+

( VLDLFC * **0.11982** )+

( His * -**0.119362** )+

( TotalFA * **0.118423** )+

( SVLDLP * **0.118397** )+

( SFA * **0.117896** )+

( Gln * -**0.117198** )+

( VLDLC * **0.112357** )+

( LHDLP * -**0.109747** )+

( VLDLP * **0.109427** )+

( DHA_A * -**0.108164** )+

( HDLCE * -**0.107923** )+

( HDLC * -**0.106626** )+

( Acetate * -**0.10631** )+

( Ile * **0.105256** );

**run**;

**data** itu; set itu;

mrs_all=

( GlycA * **0.217996** )+

( PUFAMUFA * -**0.195448** )+

( MUFA_A * **0.192041** )+

( PUFA_A * -**0.180506** )+

( Omega6_A * -**0.177534** )+

( LA_A * -**0.174586** )+

( Acetoacetate * **0.173698** )+

( Citrate * **0.164528** )+

( MUFA * **0.163087** )+

( bOHbutyrate * **0.158902** )+

( Glucose * **0.15851** )+

( XXLVLDLP * **0.150205** )+

( XLVLDLP * **0.149226** )+

( Albumin * -**0.146904** )+

( TGPG * **0.142717** )+

( TotalTG * **0.140311** )+

( LVLDLP * **0.139834** )+

( VLDLTG * **0.139651** )+

( VLDLL * **0.138936** )+

( Pyruvate * **0.138877** )+

( VLDLsize * **0.130065** )+

( VLDLPL * **0.123675** )+

( HDLsize * -**0.122485** )+

( Gly * -**0.121222** )+

( VLDLFC * **0.11982** )+

( His * -**0.119362** )+

( TotalFA * **0.118423** )+

( SVLDLP * **0.118397** )+

( SFA * **0.117896** )+

( Gln * -**0.117198** )+

( VLDLC * **0.112357** )+

( LHDLP * -**0.109747** )+

( VLDLP * **0.109427** )+

( DHA_A * -**0.108164** )+

( HDLCE * -**0.107923** )+

( HDLC * -**0.106626** )+

( Acetate * -**0.10631** )+

( Ile * **0.105256** )+

( XLHDLP * -**0.10104** )+

( VLDLCE * **0.100511** )+

( LDLTG * **0.10037** )+

( HDLTG * **0.100001** )+

( MVLDLP * **0.0995518** )+

( Unsaturation * -**0.0979918** )+

( Tyr * **0.0959632** )+

( Val * **0.0890851** )+

( ApoBApoA1 * **0.08715** )+

( Lactate * **0.0868175** )+

( TotalBCAA * **0.0855521** )+

( XSVLDLP * **0.0837005** )+

( MLDLP * **0.0834647** )+

( SHDLP * **0.0834242** )+

( LDLsize * -**0.0806329** )+

( RemnantC * **0.0758402** )+

( Acetone * **0.074707** )+

( HDLFC * -**0.0740908** );

**run**;

**data** itu; set itu;

mrs_vip_1_2=

( GlycA * **0.217996** )+

( PUFAMUFA * -**0.195448** )+

( MUFA_A * **0.192041** )+

( PUFA_A * -**0.180506** )+

( Omega6_A * -**0.177534** )+

( LA_A * -**0.174586** )+

( Acetoacetate * **0.173698** )+

( Citrate * **0.164528** )+

( MUFA * **0.163087** )+

( bOHbutyrate * **0.158902** )+

( Glucose * **0.15851** )+

( XXLVLDLP * **0.150205** )+

( XLVLDLP * **0.149226** )+

( Albumin * -**0.146904** )+

( TGPG * **0.142717** )+

( TotalTG * **0.140311** )+

( LVLDLP * **0.139834** )+

( VLDLTG * **0.139651** )+

( VLDLL * **0.138936** )+

( Pyruvate * **0.138877** )+

( VLDLsize * **0.130065** )+

( VLDLPL * **0.123675** );

**run**;

**data** itu; set itu;

mrs_vip_1_4=

( GlycA * **0.217996** )+

( PUFAMUFA * -**0.195448** )+

( MUFA_A * **0.192041** )+

( PUFA_A * -**0.180506** )+

( Omega6_A * -**0.177534** )+

( LA_A * -**0.174586** )+

( Acetoacetate * **0.173698** )+

( Citrate * **0.164528** )+

( MUFA * **0.163087** )+

( bOHbutyrate * **0.158902** )+

( Glucose * **0.15851** )+

( XXLVLDLP * **0.150205** )+

( XLVLDLP * **0.149226** )+

( Albumin * -**0.146904** );

**run**;

**PROC** **STANDARD** DATA=itu MEAN=**0** STD=**1** OUT=itu;

VAR mrs_vip1 mrs_all mrs_vip_1_2 mrs_vip_1_4; **run**;

**RUN**;
